# How to prevent viremia rebound? Evidence from a PRRSv data-supported model of immune response

**DOI:** 10.1186/s12918-018-0666-7

**Published:** 2019-01-29

**Authors:** Natacha Go, Suzanne Touzeau, Zeenath Islam, Catherine Belloc, Andrea Doeschl-Wilson

**Affiliations:** 1 BIOEPAR, INRA, Oniris, Route de Gachet, CS 40706, Nantes, France; 2 BIOCORE, Inria, INRA, CNRS, UPMC Univ Paris 06, Université Côte d’Azur, 2004 route des Lucioles, BP 93, Sophia Antipolis, France; 3 Division of Genetics and Genomics, The Roslin Institute, Easter Bush, Midlothian, UK; 4 ISA, INRA, CNRS, Université Côte d’Azur, 400 route des Chappes, BP 167, Sophia Antipolis, France

**Keywords:** Immunological model, ABC-like optimisation method, Rebounder viremia profile, PRRSv

## Abstract

**Background:**

Understanding what determines the between-host variability in infection dynamics is a key issue to better control the infection spread. In particular, pathogen clearance is desirable over rebounds for the health of the infected individual and its contact group. In this context, the Porcine Respiratory and Reproductive Syndrome virus (PRRSv) is of particular interest. Numerous studies have shown that pigs similarly infected with this highly ubiquitous virus elicit diverse response profiles. Whilst some manage to clear the virus within a few weeks, others experience prolonged infection with a rebound. Despite much speculation, the underlying mechanisms responsible for this undesirable rebound phenomenon remain unclear.

**Results:**

We aimed at identifying immune mechanisms that can reproduce and explain the rebound patterns observed in PRRSv infection using a mathematical modelling approach of the within-host dynamics. As diverse mechanisms were found to influence PRRSv infection, we established a model that details the major mechanisms and their regulations at the between-cell scale. We developed an ABC-like optimisation method to fit our model to an extensive set of experimental data, consisting of non-rebounder and rebounder viremia profiles. We compared, between both profiles, the estimated parameter values, the resulting immune dynamics and the efficacies of the underlying immune mechanisms. Exploring the influence of these mechanisms, we showed that rebound was promoted by high apoptosis, high cell infection and low cytolysis by Cytotoxic T Lymphocytes, while increasing neutralisation was very efficient to prevent rebounds.

**Conclusions:**

Our paper provides an original model of the immune response and an appropriate systematic fitting method, whose interest extends beyond PRRS infection. It gives the first mechanistic explanation for emergence of rebounds during PRRSv infection. Moreover, results suggest that vaccines or genetic selection promoting strong neutralising and cytolytic responses, ideally associated with low apoptotic activity and cell permissiveness, would prevent rebound.

**Electronic supplementary material:**

The online version of this article (10.1186/s12918-018-0666-7) contains supplementary material, which is available to authorized users.

## Background

One of the biggest challenge in infection control is dealing with heterogeneity in host response to infection. Uniphasic *vs.* multiphasic infection dynamics are of a particular interest given their potential consequences on the population dynamics and efficacies of control strategies (vaccination, genetic selection). Multiphasic infection profiles have been reported for various infections such as Influenza, HIV, Hepatitis B and C, as well as Porcine Respiratory and Reproductive Syndrome (PRRS). They can occur during natural infection (HIV [1], equine Influenza [2], PRRSv [3]), under drug therapy [4, 5] or co-infection [6, 7]. In the majority of cases, the underlying causes for multiphasic infection profiles are subject to much speculation [6, 8–12].

In this context, infection by PRRS virus (PRRSv), is of particular interest. It not only constitutes a major concern for the swine industry, responsible for significant economic losses worldwide [13, 14], but also elicits a highly diverse host response, that may contribute to the experienced difficulty in eliminating this disease despite tremendous control efforts [15–18]. Rebounders (i.e. individuals exhibiting a biphasic infection profile) have been reported for various PRRS viral strains and pig breeds[3, 11, 19, 20]. In particular, a large scale challenge experiment conducted by the Porcine Host Genetic Consortium (PHGC), in which almost 2000 pigs from various cross-breeds were infected with the same dose of a virulent PRRSv strain, revealed that around 20% of pigs exhibited viremia rebound within 6 weeks post infection, demonstrating that this phenomenon is genuine (i.e. not a simple measurement error) and common [19]. A previous study on this data set showed that the infection severity differed depending on the pig genotype; moreover, a higher proportion of rebounder pigs carried the genotype associated with severe infection [21, 22]. These results suggest that viremia rebound could be due to a genetic factor, that would lead to variable immune responses. So viremia rebound could be determined by immune mechanisms. However, mechanisms responsible for the emergence of rebound remain unclear [9, 11, 12, 19, 21, 23].

PRRSv targets antigen presenting cells (macrophages and dendritic cells), key components of the innate immune response, and hence alters the innate and the subsequent adaptive immune responses in complex ways. It induces a prolonged viremia due to its ability to hamper the whole immune response, mostly characterised by high pro-inflammatory and immuno-modulatory responses, a low antiviral response, a weak and delayed cellular response, as well as a significant but inefficient humoral response [13, 14, 24]. Moreover, infection and immune dynamics are highly variable among hosts and viral strains. Depending on experimental studies, various components of the immune response have been highlighted as having an impact on the severity and duration of PRRSv infection. The main ones are: (i) the target cell permissiveness and viral replication rate; (ii) the levels of antiviral cytokines (TNF*α*, IFN*α* and IFN*γ*) and immuno-regulatory cytokines, the latter being either pro-cellular (IL12 and IFN*γ*) or pro-humoral (IL10 and TGF*β*); (iii) the orientation of the adaptive response towards the cellular (Cytotoxic T Lymphocytes and IFN*γ*), humoral (antibodies and IL10), or regulatory (TGF*β* and IL10) response [reviews: 15–17, 25]. The aim of our study was to identify which of these immune mechanisms can reproduce and explain the rebound patterns observed in PRRSv infection dynamics. For this purpose we adopted a mechanistic modelling approach of the within-host infection dynamics.

Given the large spectrum of immune mechanisms found to influence PRRSv infection dynamics details in [26], Chap. 1, a sufficiently comprehensive representation of the multiplex immune response was required to avoid preliminary bias. This was achieved by extending an integrative model of the viral and immune component dynamics within the host representing immune mechanisms at the between-cell scale [27]. The resulting model, based on knowledge from in vitro and in vivo experimental studies on PRRSv, provides an explicit and detailed representation of: (i) the innate immune mechanisms, in particular the interactions between the virus and its target cells; (ii) the activation and orientation of the adaptive response towards the cellular, humoral or regulatory response; and (iii) the main cytokines and their complex regulations of the innate and adaptive immune mechanisms.

We fitted our within-host model to a viremia data sub-set from ([19], smoothed PHGC data). Due to the high number of model parameters, we were faced with a potential identifiability issue, preventing us from obtaining unique parameter estimates associated with each individual. However, experimental studies show that hosts challenged with the same inoculum may exhibit different immune responses and that contrasting immune responses can result in similar viremia profiles (reviewed in [26], Chap. 1). So our aim was to identify parameter sets that generate data-compatible uniphasic and biphasic viremia profiles, rather than one unique parameter set for each individual viremia profile. To do so, we developed an Approximate Bayesian Computation (ABC)-like fitting procedure, which allows an extensive exploration of a high-dimensional parameter space and is computationally less expensive than standard ABC method. This procedure resulted in the selection of two viremia sets representing the between-host variability for uniphasic and biphasic profiles respectively. We first examined the corresponding immune dynamics to characterise the response associated with the biphasic viremia profile. We then compared, between both viremia profiles, the set of estimated parameter values and the efficacies of immune mechanisms which are assumed to drive the viral dynamics. This led to the identification of discriminant candidate mechanisms, which we further explored with regards to their ability to either trigger or prevent virus load rebound. Hence, using an original model of the immune response and an appropriate systematic fitting method, the paper provides the first mechanistic explanation for PRRS viremia rebound, and possibly also for other virus infections.

## Results

**Fig. 1:**
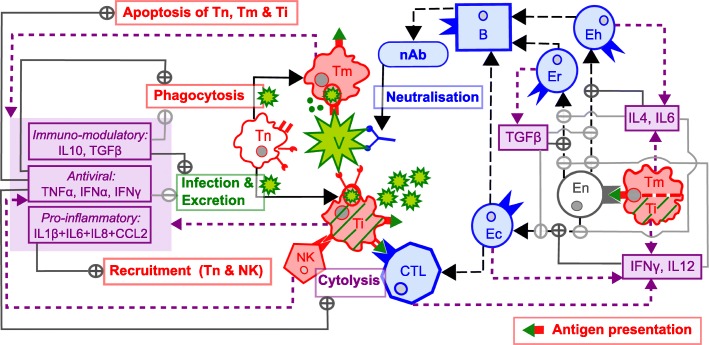
Functional diagram of the model representing the within-host immune response to PRRSv infection. Functional diagram of the model representing the within-host immune response to PRRSv infection. Binding of PRRS viral particles (*V*) and naive target cells (*T*_*n*_) either result in mature and non-infected cells (*T*_*m*_) that phagocytes viral particles, or in mature and infected cells (*T*_*i*_) that allows viral replication and excretion of new viral particles. Phagocytosis is amplified by antiviral cytokines (TNF*α*, IFN*α*, IFN*γ*) and inhibited by immuno-modulatory (IL10, TGF*β*) cytokines; on the contrary, infection and viral replication are inhibited by antiviral cytokines and amplified by immuno-modulatory cytokines. TNF*α* induces the apoptosis of *T*_*n*_, *T*_*m*_ and *T*_*i*_. Viral particles are neutralised by antibodies (nAb); infected cells are cytolysed by natural killers (NK) and Cytotoxic T Lymphocytes (CTL). Mature target cells (*T*_*m*_ and *T*_*i*_) synthesise cytokines and present the viral antigen to naive adaptive effectors (En, not explicitly represented in the model). Once activated, they differentiate into cellular (*E*_*c*_), humoral (*E*_*h*_) or regulatory (*E*_*r*_) effectors, depending on the cytokinic environment. Pro-cellular regulatory cytokines (IFN*γ*, IL12) favour *E*_*c*_, whereas pro-humoral regulatory cytokines (IL4, IL6) *E*_*h*_ and pro-regulatory regulatory cytokines (TGF*β*) *E*_*r*_. These effectors synthesise cytokines and induce the activation of plasma cells (*B*), which synthesise nAb. Moreover, *E*_*c*_ induce the activation of CTL. Finally, the recruitment of *T*_*n*_ and NK is amplified by pro-inflammatory cytokines (Pi, which groups IL1*β*, IL6, IL8 and CCL2). *Colours* – green: PRRSv particles; red: innate response; blue: adaptive response; purple: both innate and adaptive responses. *Lines* – plain with arrow: state changes; dashed (dotted) with arrow: (cytokine) syntheses; plain dark grey with ⊕: up-regulations by cytokines; plain light grey with ⊖: down-regulations by cytokines

Figure 1 shows a functional diagram of the mathematical model, which describes the evolution over time of the concentration of 19 state variables: the virus (*V*); the naive (*T*_*n*_), mature non-infected (*T*_*m*_) and mature infected (*T*_*i*_) antigen presenting cells, which are the virus target cells; the natural killers (NK); the type 1 helper T cells (cellular effectors *E*_*c*_); the type 2 helper T cells (humoral effectors *E*_*h*_); the regulatory T cells (regulatory effectors *E*_*r*_); the cytotoxic T lymphocytes (CTL); the plasma cells (*B*); the neutralising antibodies (nAb); the pro-inflammatory cytokines (Pi, which groups IL1*β*, IL6, IL8 & CCL2); the antiviral cytokines (TNF*α*, IFN*α*, IFN*γ*); the immuno-modulatory cytokines (IL10, TGF*β*); the pro-cellular regulatory cytokines (IFN*γ*, IL12); the pro-humoral regulatory cytokines (IL4, IL6); the pro-regulatory cytokine (TGF*β*).

**Fig. 2:**
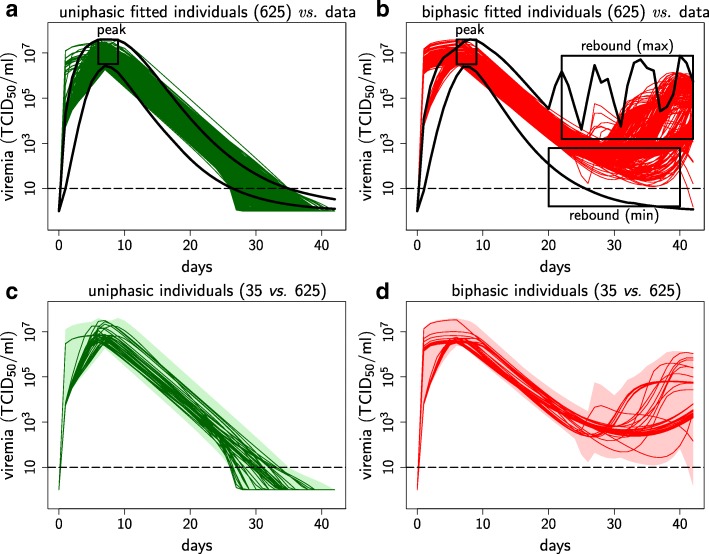
Fitted viremia over infection time for the uniphasic and biphasic profiles. Fitted viremia over infection time for the uniphasic and biphasic profiles. **a-b** Comparison between the 625 fitted individuals and the smoothed PHGC data (lower and upper envelopes in black, [19]) for the **a** uniphasic (green) and **b** biphasic (red) profiles. Black boxes: data ranges for the first viral peak, the rebound peak (max) and the minimum between the two peaks (min). **c-d** Comparison between the 35 representative individuals (lines) and the 625 fitted individuals from which the 35 were sampled (shaded area) for the **c** uniphasic (green) and **d** biphasic (red) profiles. Viremia detection threshold (horizontal dashed line). Semi-log graphs

Fitting the model to viremia data (from [19, smoothed PHGC data]) produced a wide spectrum of uniphasic and biphasic viremia profiles. For each profile, we identified 625 parameter sets, referred as “individuals”, whose viremia characteristics, i.e. infection durations, peaks and peak dates matched the viremia data (Fig. 2a & b). Differences between simulated and experimental data were observed (i) at the first few days post infection, where the model tended to predict a faster rise to peak viremia, and (ii) at the later stage of infection, where simulated biphasic profiles tended to experience a lower and later second peak than suggested by the data. Such relatively minor discrepancies are expected, given the adopted level of model complexity, and partly originate from the fact that viremia data were only sampled 8 times over 42 days, with the first sample on day 4; furthermore, experimental data were smoothed using Wood’s functions [19].

Among the 625 individuals of each profile, parameter values were not uniformly distributed. In order to capture the full range of parameters associated with each profile without sampling bias, we used a *k*-means clustering method to generate a representative sample and obtained 35 individuals per viremia profile (Fig. 2c & d, see “Selection of representative individuals for both viremia profiles” section for the clustering method).

### Characterisation of the immune response associated with the biphasic viremia profile

**Fig. 3:**
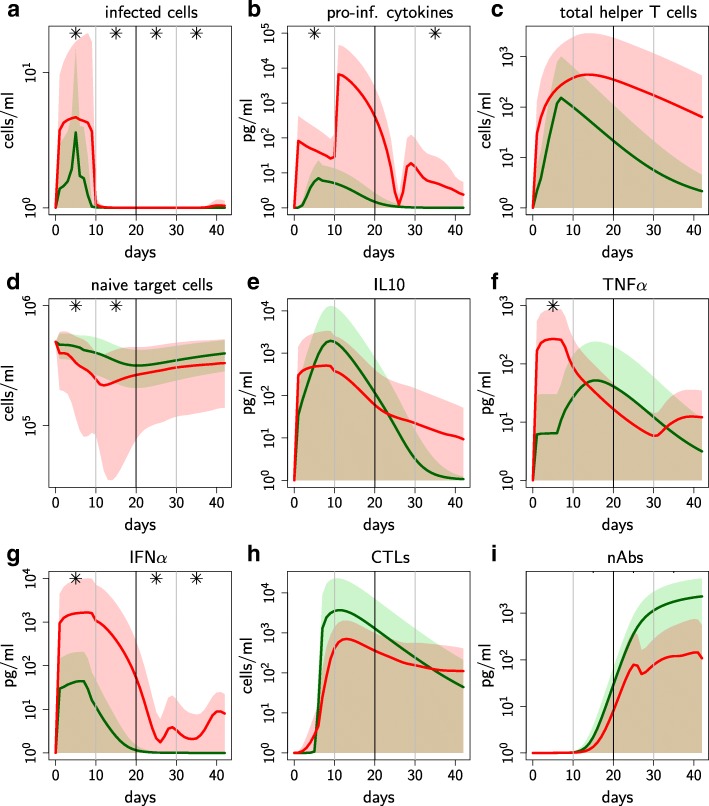
Immune components discriminating between uniphasic and biphasic viremia profiles over infection time. Immune components discriminating between uniphasic and biphasic viremia profiles over infection time. Mean value (solid line) and standard deviation (shaded area) of the 35 representative individuals selected for the uniphasic (green) and biphasic (red) viremia profiles. Semi-log graphs. ∗*p*-value <5*%* when comparing uniphasic and biphasic profiles (permutation tests over four time periods: 0–10, 11–20, 21–31, 32–42 days)

**Fig. 4:**
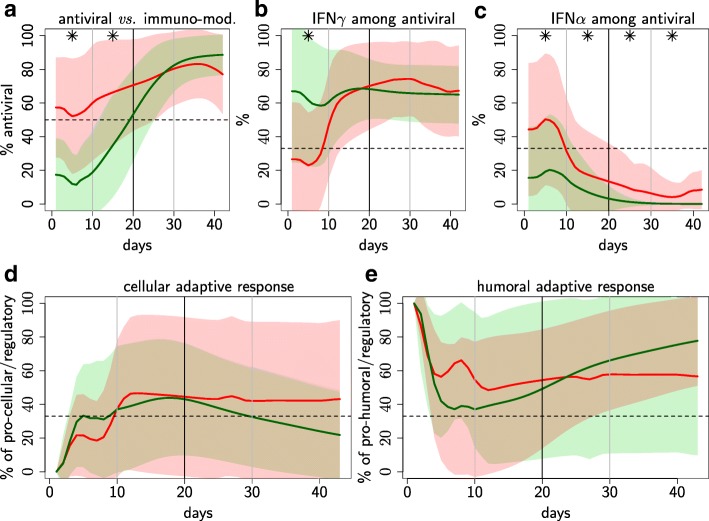
Relative cytokine levels discriminating between uniphasic and biphasic viremia profiles over infection time. Relative cytokine levels discriminating between uniphasic and biphasic viremia profiles over infection time. Percentage of **a** antiviral cytokines (IFN*γ*,IFN*α*,TNF*α*) among antiviral and immuno-modulatory (IL10,TGF*β*) cytokines; **b** IFN*γ* and **c** IFN*α* among antiviral cytokines; **d** pro-cellular (IL12,IFN*γ*) and **e** pro-humoral (IL4,Pi) cytokines over the pro-cellular, pro-humoral and pro-regulatory (TGF*β*) cytokines. Mean value (solid line) and standard deviation (shaded area) of the 35 representative individuals selected for the uniphasic (green) and biphasic (red) profiles; Balanced contribution level (horizontal dashed line). ∗*p*-value <5*%* when comparing uniphasic and biphasic profiles (permutation tests over four time periods: 0–10, 11–20, 21–31, 32–42 days)

Individuals with uniphasic and biphasic viremia profiles also had, respectively, uniphasic and biphasic profiles for most of the immune components (Additional file 1). The main characteristics that discriminate between the biphasic and uniphasic profiles, illustrated in Figs. 3 and 4, are listed below.

1 *Higher immune response activation.* The immune response activation is a global indicator of both the severity of infection and the host ability to counter infection. It is reflected by infected cell (as PRRSv targets the antigen presenting cells) and pro-inflammatory cytokine levels for the innate response, and by total helper T cell levels for the adaptive response. Biphasic profiles were associated with higher levels for these three immune components over the whole time window (Fig. 3a-c). In particular, these differences were significant for infected cells over the whole time window (Fig. 3a).

2 *Stronger depletion of naive target cells.* Infection causes a temporary reduction of naive target cells, which reduces both cell infection and immune functions of antigen-presenting cells (APC), as PRRSv targets APC. Levels of naive target cells were significantly lower for biphasic profiles until day 20 (Fig. 3d); the minimum was reached significantly earlier for biphasic profiles (test results not shown).

3 *Early predominance of antiviral vs. immuno-modulatory cytokines.* Immuno-modulatory cytokines (IL10, TGF*β*) inhibit numerous immune functions and promote the target cell permissiveness while antiviral cytokines (TNF*α*, IFN*α*, IFN*γ*) are key inhibitors of the viral multiplication. Levels of IL10 were lower for biphasic profiles until day 20 (Fig. 3e), whereas TNF*α* and IFN*α* levels were higher (Fig. 3f-g). Differences in IFN*α* levels between profiles were particularly marked over the whole time period (Fig. 3g). These results suggest a predominant antiviral response at the earlier infection stage for biphasic profiles, which was confirmed by comparing the proportion of antiviral *vs.* immuno-modulatory cytokines (Fig. 4a). Furthermore, for biphasic profiles, antiviral cytokines were initially (i.e. first week post infection) dominated by IFN*α* and then by IFN*γ*, whereas IFN*γ* always dominated for uniphasic profiles (Fig. 4b-c). Compared to IFN*α* and IFN*γ*, TNF*α* was consistently relatively low for both profiles. IL10 was the predominant immuno-modulatory cytokine for the whole infection period and for both profiles (Additional file 1 R & S).

4 *Weaker cytotoxic and neutralisation adaptive responses.* Adaptive cytotoxic response, mediated by Cytotoxic T Lymphocytes, and neutralisation response, mediated by neutralising antibodies, are key immune mechanisms to counter viral infections. Levels of cytotoxic lymphocytes were lower for biphasic profiles during almost the whole time window (Fig. 3h). Levels of neutralising antibodies were negligible for both profiles during the earlier infection stage and significantly lower for biphasic profiles from day 10 (Fig. 3i). Furthermore, the adaptive response orientation was highly variable within each profile and on average orientated towards the humoral response for both profiles over the whole time window (Fig. 4d-e).

These four immune characteristics associated with biphasic viremia profiles can result from various interacting immune mechanisms with complex cytokine regulations. For instance, depletion of naive target cells (*Characteristic 2*) can be due to low recruitment of permissive target cells controlled by pro-inflammatory cytokines, high cellular decay amplified by TNF*α*, high cell infection or phagocytosis regulated by antiviral and immuno-modulatory cytokines. Therefore, a deeper exploration into the underlying mechanisms for these discriminant immune characteristics was required.

### Identification of immune mechanisms responsible for the biphasic viremia profile

The baseline rate (i.e. model parameter,) of a given immune mechanism defines the host ability to carry out the corresponding immune function. Hence, comparing the estimated baseline rates between both viremia profiles can provide valuable information to identify the critical immune mechanisms responsible for biphasic profiles.

Nevertheless, immune components interact in complex ways involving more or less direct regulation loops via cytokines (Fig. 1). Consequently, the baseline rate (e.g. cell infection rate) does not necessarily reflect the efficacy of the mechanism (e.g. cell infection efficacy: total number of cells infected over total number of naive target cells recruited) or the dynamics of the corresponding immune component (e.g. level of infected cells over time). Therefore, the identification of critical immune mechanisms responsible for biphasic viremia profiles cannot be based on parameter values alone. Consequently, we also compared the efficacy of the mechanisms known to affect the viral dynamics directly, namely: cell infection, apoptosis and cytolysis of infected cells, as well as viral neutralisation by antibodies; or less directly: apoptosis of naive target cells.

#### Baseline rates

**Fig. 5:**
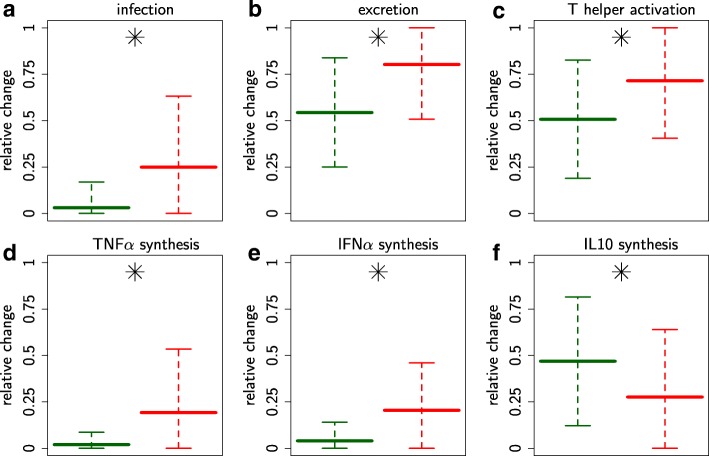
Baseline rates discriminating between uniphasic and biphasic viremia profiles. Baseline rates discriminating between uniphasic and biphasic viremia profiles. Parameters linked to **a-b** viral multiplication; **c** adaptive response activation; **d-f** antiviral (TNF*α* and IFN*α*) *vs.* immuno-modulatory (IL10) cytokine syntheses by activated target cells. Rate values are presented in relative scale, i.e. normalised according to their assumed upper and lower boundaries (see Additional file 5: Table A5-4). Mean value and standard deviation of the 35 representative individuals selected for the uniphasic (green) and biphasic (red) profiles over the parameter ranges. Parameters were significantly different between profiles (⋆*p*-value <1*%*, Kolmogorov–Smirnov test)

The values of six baseline rates among the 14 parameters estimated significantly differed between uniphasic and biphasic profiles, presented in relative scale in Fig. 5 (see Additional file 2 for all 14 parameters).

Firstly, the biphasic profile was characterised by higher baseline rates for cell infection, viral excretion and T-helper activation (Fig. 5a-c), which can explain the higher immune response activation (*Characteristic 1*) and the higher naive target cell depletion (*Characteristic 2*).

Secondly, the biphasic profile had higher baseline rates for the synthesis of TNF*α* and IFN*α* and lower baseline rates for IL10 (Fig. 5d-f), which can explain the early predominance of antiviral *vs.* immuno-modulatory cytokines (*Characteristic 3*). Moreover, as TNF*α* induces target cell apoptosis and IL10 inhibits the synthesis of TNF*α*, it can also explain the higher naive target cell depletion (*Characteristic 2*).

However, no baseline rate differences could directly explain the weaker cytotoxic and neutralisation adaptive responses for biphasic profiles (*Characteristic 4*). This characteristic probably results from multiple and indirect mechanisms.

#### Efficacies of immune mechanisms

**Fig. 6:**
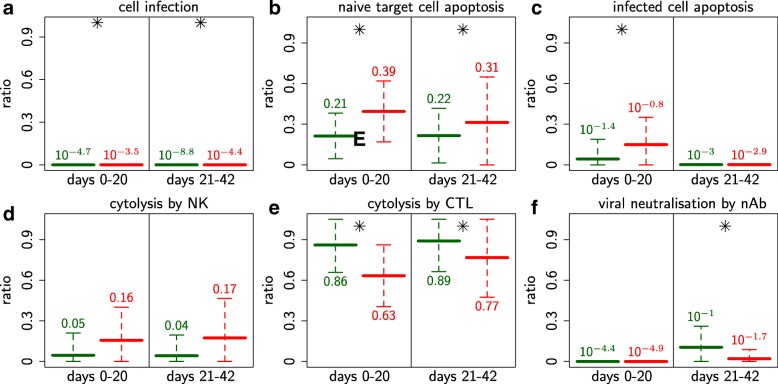
Immune mechanism efficacies discriminating between uniphasic and biphasic viremia profiles. Immune mechanism efficacies discriminating between uniphasic and biphasic viremia profiles. Efficacies of mechanisms known to affect the viral dynamics directly: **a** cell infection, **c-e** elimination of infected cells, **f** neutralisation; or indirectly: **b** apoptosis of naive target cells. Mean value and standard deviation of the 35 representative individuals selected for the uniphasic (green) and biphasic (red) profiles. ∗*p*-value <1*%* when comparing uniphasic and biphasic profiles (Kolmogorov–Smirnov tests over two time periods: 0-20, 21–42 days)

The efficacy of the key immune mechanisms (cell infection, cell, apoptosis, cytolysis of infected cells and viral neutralisation) for the uniphasic and biphasic profiles are presented in Fig. 6.

*Cell infection.* Target cell infection results in viral multiplication, but also induces the synthesis of various cytokines and the activation of the adaptive response. The infection efficacy, defined as the total number of cells infected over the total number of naive target cells recruited, was globally low (Fig. 6a). However, it was sufficient to induce host infection with realistic viremia (Fig. 2). The efficacy was significantly higher for biphasic profiles, which underpins the significant difference exhibited by the estimated infection rates. The difference between both profiles was particularly marked for the first time period, i.e. before any viremia rebound occurred. This result suggests that cell infection could be a critical immune mechanism determining viremia profile.

*Apoptosis of naive target cells.* Naive target cell apoptosis can lead to the depletion of these cells, which could be a critical mechanism to restrain cell infection. However, apoptosis efficacy, defined as the total number of naive target cells undergoing apoptosis over the total number of naive target cells recruited, was significantly higher for biphasic profiles (Fig. 6b). The difference between both profiles was particularly marked for the earlier infection stage. Apoptosis efficacy was globally high for both profiles (21 and 39% on average). These findings showed that naive target cell apoptosis was a critical mechanism and that it could determine the viremia profile.

*Elimination of infected cells.* Immune response-mediated killing of infected cells plays a fundamental role in preventing continuous production of new viral particles. Our model includes apoptosis and cytolysis as the main mechanisms that kill infected cells. Apoptosis and cytolysis efficacies were defined as the total number of infected cells undergoing apoptosis, respectively cytolysis, over the total number of cells infected. In contrast to the relatively low efficacy of apoptosis (less than 15% on average, Fig. 6c), cytolysis was found to play a major role in the destruction of infected cells for both profiles (higher than 80% on average, Fig. 6d, e).

Natural killer (NK) cytolysis efficacy was low for both profiles (4 to 17% on average) and not significantly higher for biphasic profiles (Fig. 6d) despite significantly higher levels of NK cells (Additional file 1: E). In contrast, Cytotoxic T Lymphocyte (CTL) cytolysis efficacy was high for both profiles (higher than 63% on average) and significantly lower for biphasic profiles (Fig. 6e). The difference in CTL cytolysis efficacies between both profiles was particularly marked at the earlier infection stage. This result suggests that Cytotoxic T Lymphocyte cytolysis could be a critical immune mechanism determining viremia profile, while natural killer cytolysis and infected cell apoptosis would not.

*Viral neutralisation by antibodies.* Neutralisation of viral particles by antibodies prevents new cell infection. The neutralisation efficacy, defined as the total number of viral particles neutralised over the total number of viral particles created, was low for both profiles (mean values lower than 10%, Fig. 6f). More precisely, this efficacy was almost null for both profiles at the earlier infection stage and significantly lower for the biphasic profile at the later infection stage. This result suggests that viral neutralisation is probably not a critical immune mechanism determining viremia profile.

### Validation of immune mechanisms inducing or preventing the biphasic viremia profile

**Fig. 7:**
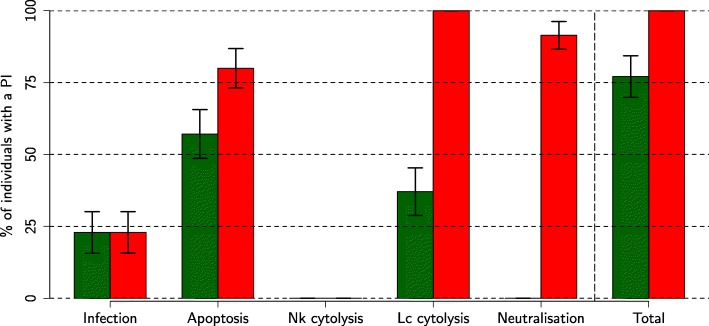
Influence of candidate immune mechanisms on viremia profile inversion. Influence of candidate immune mechanisms on viremia profile inversion. Percentage of individuals, among the 35 representative individuals selected for the uniphasic (green) and biphasic (red) viremia profiles, that had a profile inversion when boosting or inhibiting (depending on the profile) either mechanism: infection, apoptosis of naive target cells by TNF*α*, cytolysis of infected cells by NK or CTL, viral neutralisation by nAb (standard error bars were obtained by jackknifing)

In order to disentangle whether the above candidate immune mechanisms can indeed induce biphasic viremia profiles and could thus be targeted by future pharmaceutical or genetic interventions, we tested whether a viremia profile inversion could be achieved by boosting or reducing the efficacy of either one of these mechanisms. Figure 7 shows the percentages of individuals, among the 35 representative individuals selected per viremia profile, that turned from biphasic to uniphasic viremia profiles, and vice versa (Additional file 3 for more details).

Most individuals (uniphasic: 77%, biphasic: 100%) had a viremia profile inversion by varying the efficacy of at least one candidate immune mechanism. Varying the NK cytolysis efficacy never induced a viremia profile inversion. Boosting (reducing) the cell infection efficacy induced a profile inversion for almost a quarter of uniphasic (biphasic) individuals. Boosting (reducing) the apoptosis efficacy induced a profile inversion for more than half of uniphasic (biphasic) individuals. Reducing the CTL cytolysis efficacy induced a profile inversion for 37% of the uniphasic individuals, while boosting its efficacy induced a profile inversion for all biphasic individuals. Reducing the neutralisation efficacy never induced a profile inversion for uniphasic individuals, whereas boosting its efficacy resulted in a profile inversion for more than 90% of biphasic individuals.

To conclude, biphasic viremia profiles mainly resulted from high apoptosis and low CTL cytolysis efficacies; moreover, boosting CTL cytolysis or neutralisation efficacy was very efficient to prevent biphasic viremia profile.

## Discussion

Viremia rebound following a steady phase of viral decline is a common but undesirable phenomenon for PRRSv and other viral infections across a range of species [1–3, 19]. The PHGC challenge experiments, in which thousands of pigs were infected with the same dose of a virulent PRRSv strain, revealed substantial between-host variability in infection dynamics with a quarter of pigs exhibiting viremia rebound [PHGC data: 19, 19, 21]. What causes some individuals to experience viremia rebound while others manage to steadily clear the virus has however been subject to much speculation [9, 11, 12, 19, 21, 23]. Our mechanistic within-host infection model, fitted to smoothed PHGC viremia data [19], not only successfully captured the observed between-host variation in infection dynamics but also offers, for the first time, insight into potential causative immune mechanisms for generating rebound. In particular, contrary to current hypotheses emerging from genetic analyses, our model reveals that viremia rebound can occur as a result of between-host differences in the immune competence alone, without the commonly hypothesised emergence of viral escape mutants or re-infection [12, 19, 23]. This finding has profound consequences for the development of intervention strategies, as it would imply that rebound can be prevented by modifying the immune response through pharmaceuticals or genetic selection.

**Main results**

We identified several mechanisms that differed between the uniphasic and biphasic viremia profiles. Firstly, the immune response activation was higher for rebounders, although they elicited on average weaker and less efficacious cytotoxic and neutralisation responses. Rebounders also exhibited a higher cell infection efficacies, despite an early predominance of antiviral cytokines (IFN*γ*, IFN*α*, TNF*α*) over immuno-modulatory cytokines (IL10, TGF*β*). Lastly, target cell apoptosis by TNF*α* was more efficacious for rebounders, which provoked a rapid and strong depletion of naive target cells. All these differences, except for the neutralisation efficacy, occurred prior to the onset of rebound, suggesting that these were critical mechanisms that could determine viremia rebound. These results were confirmed by our validation step of the candidate immune mechanisms. Inhibiting neutralisation never generated a rebound. However, boosting infected cell cytolysis by cytotoxic T lymphocytes or viral neutralisation, as well as inhibiting target cell apoptosis, effectively prevented viremia rebound. Surprisingly, altering the efficacy of infected cell cytolysis by natural killers had no impact on the rebound.

To our knowledge, no published experimental study compared the immune response between uniphasic and biphasic PRRS viremia profiles. Mechanistic models of host response fitted to influenza virus data predicted biphasic (uniphasic) viremia profiles in the presence (absence) of IFN*α* [2, 10, 28, 29]. This finding is consistent with our results, as we showed that viremia rebounds were associated with significantly higher levels of IFN*α*.

**Modelling approach**

It should be noted that rebound patterns can be easily generated with simple models with few broad immune categories that exhibit oscillatory behaviour (see [2] for an elegant example). However, such simplistic models are generally limited in scope, are often dismissed as over-simplistic by experimental biologists, and often fail to reproduce exact patterns of real data [2, 6, 30]. The present study aimed to go a step further: our model aims to reproduce observed viremia characteristics observed in experimentally infected individuals and determine why some individuals experienced rebound while others did not.

A number of mechanistic models of virus infections in a variety of species aim at linking viremia profiles with the immune response, but only three for PRRSv [27, 31, 32]. Of particular relevance to our PRRSv modelling study are influenza models [2, 6, 10, 28–30, 33], as influenza is a respiratory virus that targets antigen presenting cells, among other cells. Moreover, viremia rebounds have been observed in natural influenza infections [2]. Influenza models often provide a fairly simplified representation of the immune response, focusing on the dynamics of few measurable immune components ([26], Chap.1) However, a recent study confronted a number of existing influenza models with experimental data and showed that these models failed to accurately reproduce at least one aspect of the immune response, even though the model parameters had been fitted to the data [10]. Moreover, several studies have pointed out the necessity of more comprehensive models to infer which immune mechanisms determine viremia characteristics [2, 6, 30].

Particularly for PRRSv, a large spectrum of immune mechanisms were found to influence the infection dynamics: target cell permissiveness, viral replication, adaptive response orientation, cytolysis, antibody neutralisation, all being modulated by various cytokines [reviews: 15–17, 25]. These mechanisms were only partly represented in the published models cited above, except in [27, 32] which provided a basis for this study but had a rougher representation of the adaptive response activation. Our study, based on an extension of these latter models, includes all relevant mechanisms and reflects the current understanding on PRRSv within-host dynamics. Our model contains an explicit and detailed representation of both the innate and adaptive immune mechanisms, including complex regulations by major cytokines, but with the minimum number of parameters. As these mechanisms are involved in most infections, this paper provides a more general framework for modelling studies requiring a representation of the global immune response.

Our holistic approach gave rise to several candidate mechanisms underlying differences in infection profiles, which are difficult to observe in experiments. Moreover, our approach illustrates an important point that is often overlooked in statistical data analysis, i.e. that differences in observed levels of immune components do not necessarily imply differences in their immune functions. For example, levels of natural killers were significantly different between both uniphasic and biphasic viremia profiles in our fitted model. However, the NK cytolysis efficacy, i.e the proportion of infected cells cytolysed by NK, was similar for both profiles. Moreover, boosting or inhibiting this efficacy neither generated nor prevented rebound. In contrast, cytotoxic T lymphocytes only differed significantly in efficacy, not in actual levels, but they were highly effective to prevent rebound.

**Fitting procedure**

A known caveat of complex models such as ours is that they inevitably require many parameters for which no prior estimates exist, thus causing a potential identifiability issue for model fitting [34]. As, on the one hand, we fitted our model only on viremia data and, on the other hand, PRRS experimental studies showed that contrasted immune responses could result in similar viremia (reviewed in [26], chapter 1), we expected our model to be non identifiable. Furthermore, the viremia data set [19] exhibits a large between-host variability within each viremia profile. Consequently, rather than reducing the model complexity and thus significantly limiting the scope of our approach, we chose to deal with this issue by relaxing the uniqueness constraint for model parameter values: we defined fitting criteria and developed a fitting procedure that identifies data-compatible parameter sets instead of one unique parameter set for each individual viremia.

For this purpose, we developed an Approximate Bayesian Computation (ABC)-like fitting procedure that extensively explores the parameter space using an Adaptive Random Search (ARS) algorithm, starting from a large number of initial conditions, This procedure allows an extensive exploration of a high-dimensional parameter space and is computationally less expensive than standard ABC. In total, over 10^9^ model simulations were performed to identify 625 data-compatible parameter sets for each viremia profile. In order to best represent the host diversity within each viremia profile, we used a clustering method to sample the 625 estimated parameter sets, rather than considering the parameter posterior distributions.

So our method does not identify parameter sets that fit individual viremia data, as many classical estimation methods do, nor does it provide posterior distributions, as Bayesian methods do. It ensures, however, that the viral indicators of the selected parameter sets match the observed data ranges. Moreover, it allowed us to overcome the identifiability issues with a reasonable computational cost and and simultaneously capture the between-host variability in individual viremia profiles.

**Comparison of model results to literature**

The fitted model not only reproduced the wide viremia range observed in the viremia data, but also generated immune response profiles similar to those reported in independent experimental studies. For example, innate immune components mainly peaked one week post infection, whereas the adaptive immune components peaked after week two [14, 35, 36] and neutralising antibodies appeared after week three [14, 24, 37]. Furthermore, cytokine levels varied substantially among simulations, with peak values in agreement with experimental observations [14, 15, 17, 24, 36, 38]. Similarly, the orientation of the adaptive response was highly variable, but on average favoured the humoral response, in line with experimental studies [15–17, 25]. Finally, our model supports experimental results that identified target cell apoptosis by TNF*α* as a critical mechanism for the early naive target cell depletion [39, 40]. Within-host dynamics selected by our fitting procedure are hence compatible with published experimental data, although we would need more longitudinal data on various immune components to fully validate our model.

However, our model is likely not an accurate representation of the early infection dynamics. Despite its enhanced level of complexity in comparison to previous PRRSv infection models, our model still constitutes a gross over-simplification of the immensely complex fine-tuned immune system. In particular, our model ignores spatial structure despite evidence that infection kinetics are tissue specific and are partly determined by migration of immune components between body compartments (e.g. [20, 41]). This is particularly important at the onset of infection, when immune cells need to be recruited to the infection site. Furthermore, the model does not incorporate time delays for immune initiation or gradual build up of immune efficacies, which are also known to play a key role in infection dynamics (e.g. [42]). These simplifications were necessary in the absence of data to inform the model parameters. However, they lead to the unrealistically sharp rises in viral load (Fig. 2) and some immune response components (Fig. 3) at the early stage of infection.

Furthermore, caution is advised when interpreting the actual estimated model parameter values (relative scales in Additional file 2 & boundaries in Additional file 5: Table A5-4,), as they are affected by several factors. For example, to limit over-parametrisation, the values of some model parameters had to be fixed to somewhat arbitrary values. As a consequence, the values of the remaining model parameters included in the fitting process partly depend on these fixed values [43]. Furthermore, as only mechanisms that had previously been identified to play a role in PRRSv infection dynamics were included in the model, the efficacy of mechanisms represented in the model could be exaggerated as these mechanisms may absorb functions of other immune mechanisms excluded from the model. Lastly, as data to inform parameter estimates for most immune components are extremely sparse in the literature, a conservatively large value range was admitted for the model parameters in both preliminary numerical explorations and fitting process. As a result of all these contributing factors, model parameter estimates may differ from their actual values. This does not affect the model conclusions, which are based on comparison between profiles that were generated under the same model assumptions.

**Alternative hypotheses for rebound**

Our study clearly illustrates that viremia rebound can originate from differences in the host (genetically regulated) immune response alone. This result appears to stand in conflict with previous genetic studies of the PHGC data, which found that viremia rebound was not heritable and which led to the conclusion that rebound was more likely caused by factors related to the virus or the environment [12, 19, 21]. So we explored the role of host immune genetics on viremia rebound further, focusing on a polymorphism (WUR SNP) previously found to confer significant differences in cumulative viremia within the first 21 days post infection [21, 22]. We found that resistant pigs, i.e. pigs carrying the beneficial allele, were less likely to experience viremia rebound (odds ratio 2.4; 95% CI: [1.2,4.9]). This implies that rebound is partly under host genetic control. The lack of genetic signal found in previous genetic analyses may potentially originate from an improper classification of pigs as rebounders or non-rebounders. Some pigs classified as non-rebounders may have experienced rebound later, i.e. outside the 42 day observation period. This is why we worked on a data subset in this study, in which we selected non-rebounders that would most probably not experience a rebound outside the observation period.

Previous studies proposed a number of alternative mechanisms responsible for the emergence of viremia rebound. These include within-host viral mutations [8, 12, 19], re-infection by infected contact individuals [6, 12] or spontaneous release of the virus from lymph nodes into the blood stream [12, 19]. Our systemic within-host model of a single strain infection cannot provide any insight into the contribution of these mechanisms to viremia rebound. However, the current model could be easily extended to test mutation and re-exposure hypotheses.

**Insights**

Our results have important implications for the development of control strategies, as they suggest that rebound could be prevented by vaccines or genetic control methods targeting specific components of the immune response. We showed that boosting the efficacy of cytotoxic T lymphocytes or neutralising antibodies in our model effectively prevented rebound. Cytotoxic T Lymphocytes and neutralising antibodies are the usual targets of vaccines [15, 17, 25, 36]. However, given the high diversity of circulating PRRSv strains, cross-protection remains a major challenge for PRRSv vaccination [15]. Consequently, vaccines using adjuvants that target non antigen-specific mechanism are particularly relevant. Interestingly, our results indicate that reducing TNF*α*-induced apoptosis should also prevent rebound, but to our knowledge, such vaccines have not yet been explored [44].

Our results also offer relevant insights for genetic disease control strategies. In particular, they suggest that marker-assisted genetic selection of pigs carrying the identified resistance allele at the WUR SNP would not only reduce the overall virus load of infected pigs anticipated from previous studies, but also reduce the occurrence of viremia rebound. It would therefore potentially help to eliminate the infection faster from the herd. Moreover, the key immune mechanisms that we identified may help to focus ongoing research about the role of the GBP5 candidate gene for this resistance SNP, which is currently poorly understood [45]. Furthermore, recent scientific breakthroughs in blocking the permissiveness of porcine alveolar macrophages to some PRRSv strains by gene editing highlight potential new avenues for genetic disease control [46, 47]. Our model suggests that considerable beneficial effects could already be achieved, even if gene editing only led to a partial reduction of target cell permissiveness.

Finally, our modelling approach, together with the model fitting and validation procedure of candidate immune mechanisms developed in this study, provides a useful template for complementing conventional statistical data analyses with more sophisticated mechanistic models. Such models integrate existing biological understanding and provide new insights into the causative underlying mechanisms of observed statistical associations. This approach can be easily adapted to other virus infections in different host species.

## Conclusion

We developed an holistic and comprehensive model of within-host PRRSv infection that represents the large spectrum of immune mechanisms influencing the infection dynamics. This model gave rise to several candidate mechanisms underlying differences in infection profiles, which are difficult to observe in experiments and can not be targeted by simplistic models. In order to identify the model parameter values that allow to generate realistic within-host dynamics, we developed an ABC-like fitting procedure. This method overcome the identifiability issues, a known caveat of such complex models, with a reasonable computational cost and a good representation of the variability among individuals. Our fitted model not only successfully capture the observed between-host variation in infection dynamics but also provide, for the first time, insight into potential causative immune mechanisms for generating PRRS viremia rebound. This finding has profound consequences for the development of intervention strategies, as it would imply that rebound can be prevented by modifying the immune response through pharmaceuticals or genetic selection.

## Methods

### Experimental data

The viremia data considered in this study were derived from longitudinal viremia measures of approximately 1600 weaner pigs experimentally infected with a virulent strain of PRRSv, carried out by the PRRS Host Genetic Consortium (PHGC^1^). A detailed description of the experimental protocol, data collection and molecular techniques can be found in [9, 48]. Briefly, the data result from a primary infection of non-isolated weaner pigs with a highly virulent North American PRRSv strain in controlled conditions, in eight distinct experimental trials (∼200 pigs per trial), carried out at the same high health farm at Kansas State University, following identical protocols. Pigs were commercial cross-breeds provided by different breeding companies, thus exhibiting a large variation in host response [19, 22]. Viremia was found moderately heritable, pointing to significant host genetic influence underlying disease severity and progression [21]. All source farms were free of PRRSv, *Mycoplasma hyopneumoniae*, and swine influenza virus. Animals were transported at weaning (average age of 21 days) to Kansas State University and randomly placed into pens of 10 to 15 pigs. After a 7-day acclimation period, pigs were experimentally infected, both intramuscularly and intranasally, with 10^5^ TCID_50_/ml of NVSL-97-7985, a highly virulent PRRSv isolate [49]. Blood samples were collected at 0, 4, 7, 11, 14, 19/21, 28, 35, and 40/42 days post infection (dpi). Serum viremia was measured using a semi-quantitative TaqMan PCR assay for PRRSv RNA. Due to the sensitivity of RT-PCR the detection threshold (and so the threshold of the infection resolution) was set at 10 TCID_50_/ml.

Visual inspection of individual viremia measures over time confirmed that all animals were infected with peak viremia levels above 10^3^ TCID_50_/ml and that only a subset of pigs (45%) had managed to clear the infection within the 42-day observation period [19, 22]. The majority of viremia data were uniphasic with a viremia peak ranging between 4 and 11 dpi, but a subset of pigs experienced a viremia rebound (viremia increase after post-peak decline) 3 weeks post infection [21]. A previous analysis showed that an adequate mathematical representation of the full range of viremia data could be obtained by fitting Wood’s functions to the longitudinal log-transformed viremia measurements of each pig, using Bayesian inference with a likelihood framework [19]. This approach not only produced for each pig a continuous smoothed viremia curve from 0 to 42 dpi, but also provided a statistical classification of individual data into non-rebounders with a uniphasic viremia profile and rebounders with a biphasic viremia profile [19]. Based on this analysis, 17% of the data were classified as biphasic, indicating that viremia rebound is a genuine phenomenon rather than a measurement error.

**Subset selection**

In this study, we aimed at identifying the immune mechanisms that discriminate between uniphasic and biphasic viremia profiles, from data observed during a 42-day observation period. To do so, we selected a relevant subset of the smoothed PHGC data [19]. Firstly, we needed to ensure that the uniphasic data would most probably not exhibit a second viremia peak beyond the 42-day observation period. So we only considered data showing a clear resolution of viremia, i.e. viremia measurements below 10 TCID_50_/ml, the detection threshold, over at least two consecutive weeks within 35 dpi. This more constraining criterion, rather than 42 dpi, was chosen based on the observation that no viremia curve exhibiting 7 consecutive days or more below the detection threshold was classified as biphasic ([19], personal communication). With this first constraint, 20% of the curves initially classified as uniphasic were retained.

**Fig. 8:**
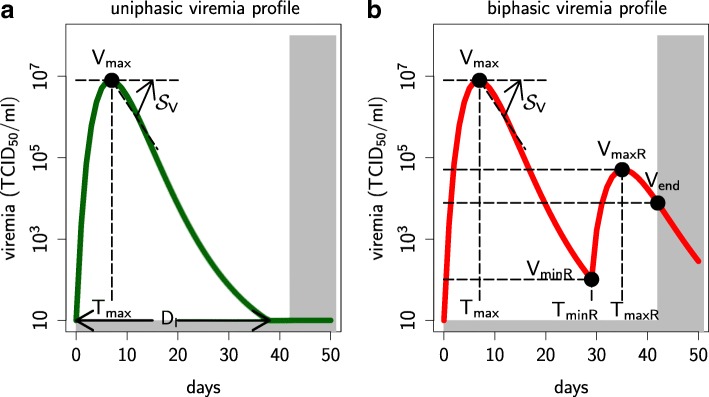
Definition of viral indicators for the uniphasic and biphasic profiles. Definition of viral indicators for the uniphasic and biphasic profiles. For both profiles **a**-**b**: viral peak (*V*_max_), date of viral peak (*T*_max_), standardised rate of viremia decline after the peak ($\mathcal {S}_{V}$, defined in Eq. (1)) and infection duration (*D*_*I*_) when defined, i.e. when the viremia remains under the detection threshold until the end of the experiment. For the biphasic profile only **b**: minimum reached before the second viral peak (*V*_minR_), date at which this minimum is reached (*T*_minR_), second viral peak (*V*_maxR_), date of second viral peak (*T*_maxR_), viral titer at the end of the experiment *V*_end_ when defined, i.e. when the viremia is above the detection threshold. Grey area: viremia lower than the detection threshold (10 TCID_50_/ml) or after the end of the experiment

Secondly, we wanted the uniphasic and biphasic profiles to be as comparable as possible during the phase corresponding to the first viremia peak, i.e. prior to the rebound onset (0 to 20 dpi). Previous analyses of the complete data set found no significant differences between the two profiles within the first 21 dpi [19]. To reinforce their similarity, we added extra constraints on the following three key profile shape indicators (Fig. 8): the viral peak (*V*_max_), the date of the viral peak (*T*_max_) and the standardised viremia decline rate after the peak ($\mathcal {S}_{V}$). The latter was defined as follows (with *V*(*t*) the viral titer over time *t*):

1 $$ \begin{aligned} \mathcal{S}_{V} =& \frac{V_{\text{max}}-V(t=T_{\text{max}}+12)}{12 \times V_{\text{max}}},  \end{aligned}  $$

**Fig. 9:**
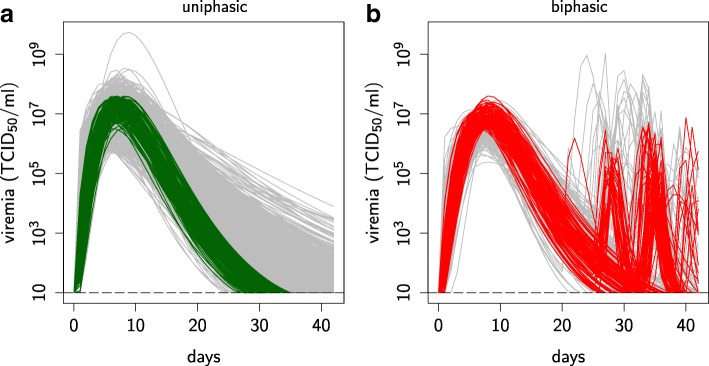
Uniphasic and biphasic viremia data subsets. Uniphasic and biphasic viremia data subsets. Selection from ([19], smoothed PHGC data) of **a** 131 (green curves) out of 1091 pigs for the uniphasic profile and **b** 109 (red curves) pigs out of 227 pigs for the biphasic profile (non selected data in grey). Data smoothed by fitting Wood’s functions [19]. Dotted line: detection threshold

The 12-day post-peak time chosen in Eq. (1) corresponds more or less to half the peak value for uniphasic curves; moreover, it always precedes the rebound onset for biphasic curves. We then selected curves with key indicators within the range shared by the uniphasic data preselected in the first step described above and the biphasic data (Table 1). This second and final step resulted in a subset of 131 non-rebounders, representing 12% of all uniphasic data, and 109 rebounders, representing 48% of all biphasic data. Selected data are illustrated in Fig. 9 and provided in Additional file 4.

**Table 1:** Summary statistics (minimal min., mean and maximal max. values) of the viral indicators (defined in Fig. 8) for the uniphasic (131 pigs) and biphasic (109 pigs) viremia data subsets (from [19, smoothed PHGC data])

Indicators	*V*_max_	*T*_max_	$\mathcal {S}_{V}$	*V*_minR_	*T*_minR_	*V*_maxR_	*T*_maxR_	*D*_*I*_	*V*_end_
[units]	[V]	[days]	[days ^−1^]	[V]	[days]	[V]	[days]	[days]	[days]
*Uniphasic*									
min.	6.5	6.0	0.032	–	–	–	–	27	–
mean	7.2	7.1	0.038	–	–	–	–	33	–
max.	7.6	9.0	0.048	–	–	–	–	35	–
*Biphasic*									
min.	6.5	6.0	0.031	<1	20	3.2	22	31	–
mean	6.9	7.6	0.038	1.2	31	5.0	33	37	–
max.	7.6	9.0	0.049	2.8	40	6.9	42	^⋆^	5.7

[V] viremia unit in log: logTCID_50_/ml

^⋆^12 out of 109 data curves were not resolved at 42 dpi (*V*_end_>1)

### Mathematical model

We used a deterministic model that describes the within-host dynamics induced by a primary PRRSv infection in a PRRSv-naive post-weaning pig. The model represents the mechanisms at the between-cell scale and provides an integrative view of the immune response. It extends a previous model representing the dynamics in the main infection place, the lung, which allowed to identify the immune mechanisms that determine the infection duration [27]. This previous model focused on the interactions between the virus and its main target cells in the lung, the pulmonary macrophages, which are major cells of the innate immune system. It included an explicit and detailed description of the innate immune response and a coarse description of the adaptive response, in addition to the main cytokines and their complex regulations of the immune mechanisms. Compared to this previous model, we mainly detailed the activation and orientation steps of the adaptive response in order to get a more balanced and realistic view of the immune response in the whole pig.

The model describes the evolution over time of the concentration of 19 state variables: the virus, three states for the target cells, the natural killers, five types of effector cells of the adaptive response, the neutralising antibodies, and eight (groups of) cytokines. The functional diagram of the model is shown in Fig. 1. Our modelling assumptions are detailed and justified in Additional file 5, which gives a complete description of the model and corresponding equations. We present below an outline of the model, detailing a few representative key processes and equations, with an emphasis on the adaptive response.

**Viral particles and target cells**

PRRS viral particles (*V*) target antigen-presenting cells, consisting of alveolar macrophages, conventional and plasmacytoid dendritic cells. These cells are represented as a functional group with three states: naive (*T*_*n*_), mature and non-infected (*T*_*m*_), or mature and infected (*T*_*i*_).

The infection is initiated by the influx of viral particles into the infection site, represented by an exposure function of time *E*(*t*) mimicking the infection protocol [32]. The interaction between viral particles and naive or mature target cells either results in cell infection (rate *β*_*T*_) or phagocytosis of viral particles (rate *η*_*T*_). These interactions are regulated by cytokines, that either activate *κ*^+^(∙), amplify: 1+*κ*^+^(∙), or inhibit: *κ*^−^(∙) a mechanism. Phagocytosis is amplified by antiviral cytokines (TNF*α*, IFN*α*, IFN*γ*) and inhibited by immuno-modulatory cytokines (IL10, TGF*β*); infection regulations are just the opposite. Cell infection results in the excretion of free viral particles (rate *e*_*T*_), representing the replication within the cell and release outside the cell, which is inhibited by antiviral cytokines. Viral particles are subject to natural decay (rate $\mu _{V}^{\text {nat}}$) and can be neutralised by antibodies nAb (rate $\mu ^{\text {ad}}_{V}$). The resulting viral dynamics, which determines the viremia profiles (as those depicted in Fig. 9), is formalised in the Eq. 2.


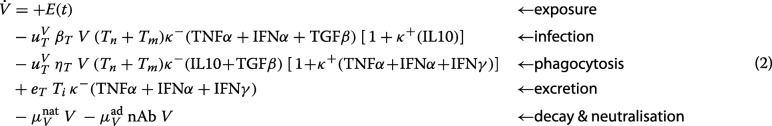


Recruitment of naive target cells *T*_*n*_ to the infected site (rate *R*_*T*_) is amplified by pro-inflammatory cytokines Pi (grouping IL1*β*, IL6, IL8 and CCL2) and IL12 acting in synergy. Through phagocytosis (*T*_*n*_ become *T*_*m*_) or infection (*T*_*n*_ become *T*_*i*_), naive target cells are activated and become mature cells. Mature non-infected cells (*T*_*m*_) eventually revert to the naive state (rate *γ*_*T*_). This activation loss is amplified by immuno-modulatory cytokines. In addition to natural decay (rate $\mu _{T}^{\text {nat}}$), TNF*α* induces the apoptosis of target cells (rate $\mu ^{\text {ap}}_{T}$). The resulting dynamics of naive target cells is formalised in the Eq. 3.


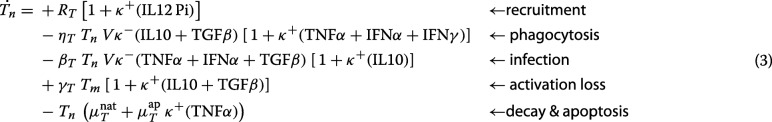


Similar equations depict the dynamics of infected and mature non-infected (which can be infected) target cells. They include an extra feature: the cytolysis of infected cells by natural killers (NK) and Cytotoxic T Lymphocytes (CTL).

**Innate immune response**

The virus target cells pertain to the innate response. Mature target cells are involved in the virus phagocytosis and in antigen presentation, which activates the adaptive response. The model also includes another innate cell type, the activated natural killers (NK), which cytolyse infected cells. All these cells synthesise various cytokines.

**Adaptive immune response**

The first step of the adaptive response is the activation (rates $\alpha _{E}^{T_{m},T_{i}}$) of helper T cells by mature antigen-presenting target cells (*T*_*m*_ and *T*_*i*_). Depending on the cytokine environment, they differentiate into the three main CD$^{+}_{4}$ T lymphocyte subtypes, that determine the adaptive response orientation: the cellular (*E*_*c*_, type-1 helper T cells), humoral (*E*_*h*_, type-2 helper T cells) and regulatory (*E*_*r*_, regulatory and type-17 helper T cells) effectors. The humoral subtype is the default and remains so when cytokines IL4 and IL6 (in Pi) predominate over IL12, IFN*γ* and TGF*β*. The dynamics of the humoral effectors appears in the following in the Eq. 4.


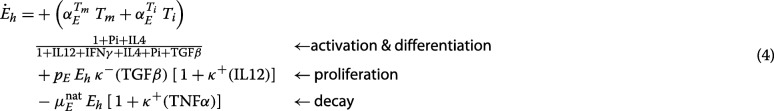


The equations of the cellular and regulatory effectors are similar, except for the differentiation term: IL12 and IFN*γ* favour the cellular response, TGF*β* the regulatory response.

Once differentiated, together with viral particles, these three effectors activate plasma cells (*B*, rate $\alpha _{B}^{E}$), which in turn synthesise neutralising antibodies (rate $\rho ^{B}_{\text {nAb}}$, see Eq. 5.

5 $$ \begin{aligned} \dot B &= + \alpha_{B}^{E}\,\textstyle\frac{V}{1+V} \left(E_{h} + E_{c} + E_{r}\right) &\quad \leftarrow &\textsf{activation}\\ &\quad + p_{B}\,B\,{\kappa^{-}(\text{TGF}\beta)}&\quad \leftarrow &\textsf{proliferation}\\ &\quad- \mu_{B}^{\text{nat}}\,B &\quad \leftarrow &\textsf{decay}\\ \end{aligned}  $$

Moreover, the cellular effectors (*E*_*c*_), together with mature target cells, activate CD$^{+}_{8}$ T cells, also called Cytotoxic T Lymphocytes (CTL, rates $\alpha _{\text {CTL}}^{T_{m},T_{i}}$), which induce the cytolysis of infected cells.:

6 $$ {\begin{aligned} \dot{\text{CTL}} &\!=+ \textstyle\sum_{j=m,i}\left(\alpha_{\text{CTL}}^{T_{j}}\,\frac{T_{j}}{1+T_{j}}\right)\,E_{c}&\; \leftarrow &\textsf{activation}\\ &\quad+p_{E}\,\!\text{CTL}\,{\kappa^{-}\!(\text{TGF}\beta)}}{[1+\kappa^{+}(\text{IL}12)]&\; \leftarrow &\textsf{proliferation}\\ &\quad- \mu_{\text{CTL}}^{\text{nat}}\,\text{CTL}}\,{[1+\kappa^{+}(\text{TNF}\alpha)]&\; \leftarrow &\textsf{decay}\\ \end{aligned}}  $$

Proliferation of all five adaptive effectors (rates *p*_*E*_ and *p*_*B*_) is inhibited by TGF*β* and amplified by IL12 (except for *B*). Their natural decay (rates $\mu _{.}^{\text {nat}}$) is amplified by TNF*α* (except for *B*), which induces their apoptosis. The effectors synthesise various cytokines.

**Cytokine regulations**

The model accounts for eight cytokines, representing the major cytokines functions: pro-inflammatory (Pi, grouping IL1*β*, IL6, IL8 and CCL2), antiviral (TNF*α*, IFN*α* & IFN*γ*), immuno-modulatory (IL10 & TGF*β*) and immuno-regulatory, the latter being subdivided in pro-cellular (IL12 & IFN*γ*), pro-humoral (IL4 & IL6) and pro-regulatory (TGF*β*) responses.

Cytokines are synthesised by the immune cells. They are involved in the regulation of most infection and immune processes, including the cytokine syntheses. Up-regulations, whether activations (multiply by *κ*^+^) or amplifications (multiply by [1+*κ*^+^]), and down-regulations (multiply by *κ*^−^) depend on the cytokine concentration (*C*_*k*_). The higher the cytokine concentration, the stronger the effect. However, there is a limited number of receptors, so the effect saturates above a given cytokine concentration. Cytokine effects are hence classically based on the Michaelis–Menten formalism [50–52]:

7 $$ \kappa^{+}(C_{k}) = \frac{v_{m} \: C_{k}}{k_{m}+C_{k}} \quad \& \quad \kappa^{-}(C_{k}) = \frac{k_{m}}{k_{m}+C_{k}},  $$

where *v*_*m*_ denotes the saturation factor and *k*_*m*_ the half saturation constant. Cytokines may interact: an additive effect of cytokines *C*_*k*_ and *Ck*′ is represented by *κ*^±^(*C*_*k*_+*Ck*′), a synergistic effect by *κ*^±^(*C*_*k*_ *Ck*′). To reduce the model complexity, we assumed that the regulation parameters *k*_*m*_ and *v*_*m*_ were the same, whatever the cytokine(s) involved. This simplification was based on our sensitivity analyses, where both parameters were found to exhibit a negligible influence on the viral dynamics (Additional file 5: Figure A5-1).

### Model fitting

The observed infection dynamics varies considerably among individuals (Fig. 9). We hypothesised that the variability within and between the uniphasic and biphasic viremia profiles is related to a different balance among immune mechanisms and that it can be captured by our mechanistic model using various parameter sets. In order to identify parameter sets associated with either the uniphasic or the biphasic profile, we fitted the model to the experimental data subsets. Since the model has many parameters, we were faced with a potential identifiability issue for obtaining unique parameter estimates associated with a specific profile. Rather than reducing the model complexity and thus significantly limiting the scope of our approach, we first reduced the number of parameters to estimate and, second, chose an appropriate fitting procedure.

#### Parameter ranges and selection

There are few experimental or modelling data to inform the parameter values. In previous work [26, 27], we developed a specific procedure to tackle this issue: (i) similar models in the literature provided large ranges for model parameters; (ii) quantitative (for the viremia), semi-quantitative (orders of magnitude, date of peaks, *etc.* – for immune components) and qualitative (shape – for immune components) data from PRRSv experimental studies were collected to define realistic within-host dynamics; (iii) through an extensive exploration of the parameter space, parameter ranges were refined to obtain realistic dynamics. We hence obtained ranges for all model parameters (Additional file 5: Table A5-4 & Figure A5-2).

To select which parameters to estimate and which to fix, we performed global sensitivity analyses on the whole viral dynamics (Additional file 5). A first sensitivity analysis exploring the influence of (almost) all model parameters exhibited that those parameters had comparable contributions to the viremia variance, so we could not identify a subset of parameters with a marked influence on the viremia.

Consequently, we based our parameter selection on biological knowledge. Hypotheses linking between-host variability in the infection dynamics to immune mechanisms are numerous [15–18, 25, 53]. In order to remain open to all these hypotheses, we selected 14 parameters that are associated with a wide range of relevant mechanisms: the virus capacity to infect the cell and replicate, the target cell capacity to synthesise antiviral *vs.* immuno-modulatory cytokines, and the activation of the different arms of the adaptive response. To avoid biasing our results, the ranges of the 14 parameters to estimate were set equally for both profiles.

Fixing the remaining parameters to an intermediate value of their respective ranges, we performed a second sensitivity analysis. As previously, it could not single out parameters with a major impact on the viremia, but the exploration of the parameter subspace showed that we covered more than the variability observed in viremia data.

#### Fitting procedure

We estimated the parameter values associated with each profile by minimising a criterion which quantifies the distance between a model simulation and the data. Our aim was to identify parameter sets that characterise each viremia profile. Rather than reproducing individual viremia data, as classical fitting criteria do, we defined, for each profile, a criterion based on the whole data range. A data-compatible parameter set was then defined as a set that satisfies (minimises) this criterion. We then looked for not one but several data-compatible parameter sets. To do so, we developed a fitting method that resembles Approximate Bayesian Computation (ABC), but is less computationally costly. This allowed us to specify a full range of data-compatible parameter sets.

*Fitting criteria.* Before calculating the fitting criterion, the simulation profile was determined. A viremia curve was classified as: (i) *uniphasic* if and only if (i) it exhibited a single peak above the detection threshold within the first 42 days of infection and (ii) the viremia was below the detection threshold at day 42; (ii) *biphasic* if and only if it exhibited at least two peaks above the detection threshold within the first 42 days of infection.

If the simulated viremia curve matched the expected profile, the corresponding criterion was computed as follows. Both uniphasic and biphasic criteria were based on the viral indicators (Fig. 8) and the corresponding data ranges (Table 1), rather than the whole viremia dynamics. For each indicator *i*, the error *Δ*_*i*_ was defined as the shortest standardised distance between the viral indicator value simulated by the model $\mathcal {I}^{M}_{i}$ and the corresponding range observed in the data set $\left [\mathcal {I}^{\min }_{i}, \mathcal {I}^{\max }_{i}\right ]$. The fitting criterion ($\mathcal {C}$) was then defined as the sum of squared errors of the relevant viral indicators (*n*=4 for the uniphasic profile, *n*=9 for the biphasic profile):

8 $$ \begin{aligned}\mathcal{C}&=\sum\limits_{i=1}^{n}\Delta_{i}^{2}\\ &\quad \text{with:}\\ \Delta_{i} &= \left\{ \begin{array}{ll} 0 & \text{if }\mathcal{I}^{M}_{i}\in \left[\mathcal{I}^{\min}_{i},\mathcal{I}^{\max}_{i}\right], \\ \frac{\min\left(|\mathcal{I}^{M}_{i}-\mathcal{I}^{\min}_{i}|, |\mathcal{I}^{M}_{i}- \mathcal{I}^{\max}_{i}|\right)}{\left(\mathcal{I}^{\max}_{i}-\mathcal{I}^{\min}_{i}\right)} &\text{else.} \end{array}\right. \end{aligned}  $$

Indicator errors were normalised to account for differences in terms of magnitude and ranges. As we aimed for zero-valued errors for all indicators, if some indicator errors had outweighed the others, it could have affected the convergence of the optimisation algorithm. NB: Viral indicators would correspond to the summary statistics in an ABC method. A fitting criterion equal to zero would correspond to an ABC acceptance criterion with: (i) ABC data defined as mean viral indicator values; (ii) ABC tolerance defined as half the viral indicator ranges.

If, in contrast, the simulated viremia curve did not match the expected profile, the corresponding fitting criterion $\mathcal {C}$ was set to an arbitrarily high value, in order to penalise the corresponding parameter set. If the viremia curve matched a biphasic (resp. uniphasic) profile when a uniphasic (resp. biphasic) was expected, we set $\mathcal {C}=500$. If the viremia curve exhibited a single viral peak but was unresolved at day 42, we set $\mathcal {C}=700$. Finally, if the viremia curve did not exhibit any peak (either a steady growth or unsuccessful infection), we set $\mathcal {C}=1000$.

*Implementation and initialisation.* The model simulation and model fitting were conducted in Scilab 5.5.3 [54]. The minimisation, i.e. the identification of data-compatible parameter sets resulting in $\mathcal {C}=0$ (8), was performed using the Adaptive Random Search (ARS) algorithm, for both uniphasic and biphasic profiles independently. ARS is a simple optimisation method, exhibiting good empirical performance: numerous case studies have demonstrated that the algorithm searches efficiently through large and complex search spaces before reaching the perceived global optimum [55], i.e. $\mathcal {C}=0$ (8) in our case.

In order to thoroughly explore the parameter space, we performed the fitting procedure starting from 625 initial parameter sets that proceeded independently. They were chosen following a fractional factorial design built with the R package *planor* [56], in order to distribute the algorithm starting points evenly in the parameter space, minimising the computational effort. This method is particularly well suited for high dimensional problems characterised by multiple influential parameters with strong interactions, as was the case in our study (see Additional file 5). 625 corresponds to the number of points required for a resolution IV design with 3 levels per parameter. For each parameter set staring point, the ARS algorithm converged to an optimal parameter set, corresponding to $\mathcal {C}=0$ (8), within 10^5.8^ iterations on average.

### Analyses

*Selection of representative individuals for both viremia profiles.* In order to capture the full range of parameter combinations associated with each profile without sampling bias, we generated a representative set of 35 individuals per viremia profile using a clustering method. Indeed, among the 625 individuals of each profile, some were very close, others quite distinct. Consequently, taking into account the full set would have lead to over- or under-representations of some individuals. We proceeded similarly but independently for both viremia profiles. We used a k-means clustering method (kmeans function of R package stats), which partitions the 625 parameter sets obtained by the fitting procedure into a given number of clusters. The number of clusters has to be big enough in order to represent the variability of the parameter sets but small enough to prevent the over-representation of some parameter sets. To set the number of clusters, various heuristics are employed, such as the “elbow rule”: between-class inertia increasing with the number of clusters, this rule consists in detecting the cluster number for which adding another cluster does not result in a notable inertia increase. We ran the k-means method for all possible numbers of clusters (1 to 625) and used the “elbow rule” to get a rough idea of the appropriate number of clusters (between 20 and 50). We then selected the smallest number corresponding to 80% between-class inertia for both profiles, namely 35 clusters. The representative individual of each cluster was chosen as the parameter set closest to the cluster barycentre. 35 individuals over the 625 represent a sufficiently large number to cover the full range of parameter combinations associated with each profile and to provide sufficient statistical power to detect differences between both profiles.

*Identification of immune mechanisms discriminating between both viremia profiles.* In order to identify the key immunological drivers that lead to either uniphasic or biphasic viremia profiles, we compared, for the 35 individuals selected per profile: (i) the dynamics of the immune components represented in the model, over four time periods (0-10, 11–20, 21–31, 32–42 dpi); (ii) the estimated baseline rates of the immune mechanisms; and (iii) the efficacy of key immune mechanisms for the earlier (0 to 20 dpi) and later (21 to 42 dpi) time periods. The efficacy of a particular immune mechanism (e.g. cell infection, viral neutralisation, infected cell cytolysis, etc.) was quantified by the ratio of the total number of viral particles or cells mobilised by the mechanism and the total number of viral particles or cells generated, over the time period considered. As an example, the efficacy of viral neutralisation was defined, from Eq. 2, as the total number of viral particles neutralised $\: \int _{t=t1}^{t2} \mu _{V}^{\text {ad}}\: V(t)\: \text {nAb}(t) \: dt$ over the total number of viral particles created (exposure + replication): $\int _{t=t1}^{t2} e_{T} \: T_{i}(t)\:\kappa ^{-}(\text {TNF}\alpha (t)+\text {IFN}\alpha (t)+\text {IFN}\gamma (t))\:+ \:E(t) \: dt$.

Comparisons were performed by visual inspection and standard uni-variate statistical tests: mean values and standard deviations, permutation tests for the trajectories (R package stamod, with 10^4^ permutations) and Kolmogorov–Smirnov for the baseline rates and mechanism efficacies (R package Matching).

**Validation of candidate immune mechanisms**

Viremia profiles are the result of many immune mechanisms that interact and are regulated by complex feedback loops. However, pharmaceutic interventions can often only target a single mechanism. We therefore tested whether boosting or inhibiting specific key mechanisms could result in a viremia profile inversion, e.g. from uniphasic to biphasic or vice versa.

For this purpose, we carried out additional simulations in which we boosted (resp. inhibited) the values of the parameters driving the efficacy of each mechanism by six gradual levels from ×10 (10^−1^) to ×10^3^ (resp. 10^−3^). We performed the simulations on the 35 representative individuals per viremia profile. We focused on mechanisms which directly affect the viral dynamics and are assumed to play a critical role in the infection dynamics [17, 57, 58]: infection (driven by infection rate *β*_*T*_), naive target cell apoptosis by TNF*α* (apoptosis rate $\mu _{T}^{\text {ap}}$), infected cytolysis by natural killers and cytotoxic T lymphocytes (cytolysis rates $\mu _{T}^{\text {in}}$ and $\mu _{T}^{\text {ad}}$), viral particle neutralisation by antibodies (neutralisation rate $\mu _{V}^{\text {ad}}$). When a mechanism exhibited a higher (lower) efficacy for the viremia profile of the individual, we decreased (boosted) its efficacy. Then we counted the percentage of individuals that had a profile inversion for at least one of the six levels tested. A simulation of a uniphasic (resp. biphasic) individual qualified for profile inversion if the corresponding viral titer could be classified as biphasic (resp. uniphasic) according to the definition of the viremia profile given in the “Fitting criteria” paragraph above.

**Role of host genetics on rebound**

Genetic studies of the PHGC data identified a single nucleotide polymorphism (WUR10000125) on chromosome 4, denoted WUR SNP, that was found to confer significant differences in cumulative viremia (i.e. area under the viremia curve) within the first 21 days post infection [21, 22]. So we tested whether the WUR SNP was also associated with rebound. For this purpose, we carried out a logistic regression analysis on our data subset. We categorised pigs into two resistance genotypes, high and low, according to whether or not they carried the beneficial allele at the WUR SNP. We accounted for systematic effects in this analysis [19, 21]. The result of this analysis is presented in the Discussion alone.

## Additional files

Additional file 1 Within-host dynamics. Evolution over infection time of the 19 state variables of the model: free viral particles, target cells (APC), natural killers, adaptive effectors including cytotoxic T lymphocytes, plasma cells, neutralising antibodies and cytokines. Comparison between uniphasic and biphasic viremia profiles. (PDF 124 kb)

Additional file 2 Estimated parameters. Values of the 14 parameters linked to between-host variability: host–virus interactions, viral replication, activation of the adaptive response, cytokine syntheses by activated target cells; for the 35 representative individuals selected for the uniphasic and biphasic viremia profiles. Comparison between uniphasic and biphasic viremia profiles. (PDF 87 kb)

Additional file 3 Mechanisms resulting in viremia profile inversion. Percentage of individuals, among the 35 representative individuals selected for the uniphasic and biphasic viremia profiles, that had a profile inversion when boosting or inhibiting (depending on the profile) either mechanism (one at a time): infection, apoptosis by TNF*α*, cytolysis by cytotoxic T lymphocytes, neutralisation by antibodies. Cytolysis by natural killers never resulted in a profile inversion (not shown). (PDF 87 kb)

Additional file 4 Smoothed viremia data. Parameters of the Wood’s functions fitted to the PHGC data [19], for the selected individuals exhibiting uniphasic or biphasic profiles. (PDF 121 kb)

Additional file 5 Model description & Sensitivity analyses. The file provides a complete description of the dynamic model representing the within-host dynamics induced by a primary PRRSv infection in a naive pig. It specifies the modelling assumptions and includes all model equations. The file also describes the global sensitivity analyses performed to assess the impact of model parameters on the viral dynamics. Corresponding aims, methods and results are presented. (PDF 710 kb)

## Abbreviations

ABC: Approximate bayesian computation
APC: Antigen presenting cell
ARS: Adaptive random search
CI: Confidence interval
CTL: Cytotoxic t lymphocyte
dpi: Days post-infection
HIV: Human immunodeficiency virus
nAb: Neutralising antibody
NK: Natural killer
PCR: Polymerase chain reaction
PHGC: Porcine host genetic consortium
PRRSv: Porcine reproductive and respiratory syndrome virus
RNA: Ribonucleic acid
TCID_50_/ml: 50% Tissue culture infective dose
WUR SNP: Single nucleotide polymorphism WUR10000125

## Footnotes

1 PHGC: http://www.animalgenome.org/lunney/index.php
