# Supplementary material for: How to prevent viremia rebound? Evidence from a PRRSv data-supported model of immune response
Source: BMC Syst Biol. 2019 Jan 29;13:15. doi: 10.1186/s12918-018-0666-7 (PMC6352383; doi:10.1186/s12918-018-0666-7)

The figure provides evolution over infection time of the 19 state variables of the model: **A** free viral particles, **B-D** target cells (APC), **E** natural killers, **F-I** adaptive effectors including **G** cytotoxic T lymphocytes, **J** plasma cells, **K** neutralising antibodies and **L-S** cytokines. Mean value (solid line) and standard deviation (shaded area) of the 35 representative individuals selected for the uniphasic (green) and biphasic (red) viremia profiles (semi-log graphs).
$*$ p-value$<$ 1% when comparing uniphasic and biphasic profiles (permutation tests over four time periods: 0-10, 11-20, 21-31, 32-42 days).

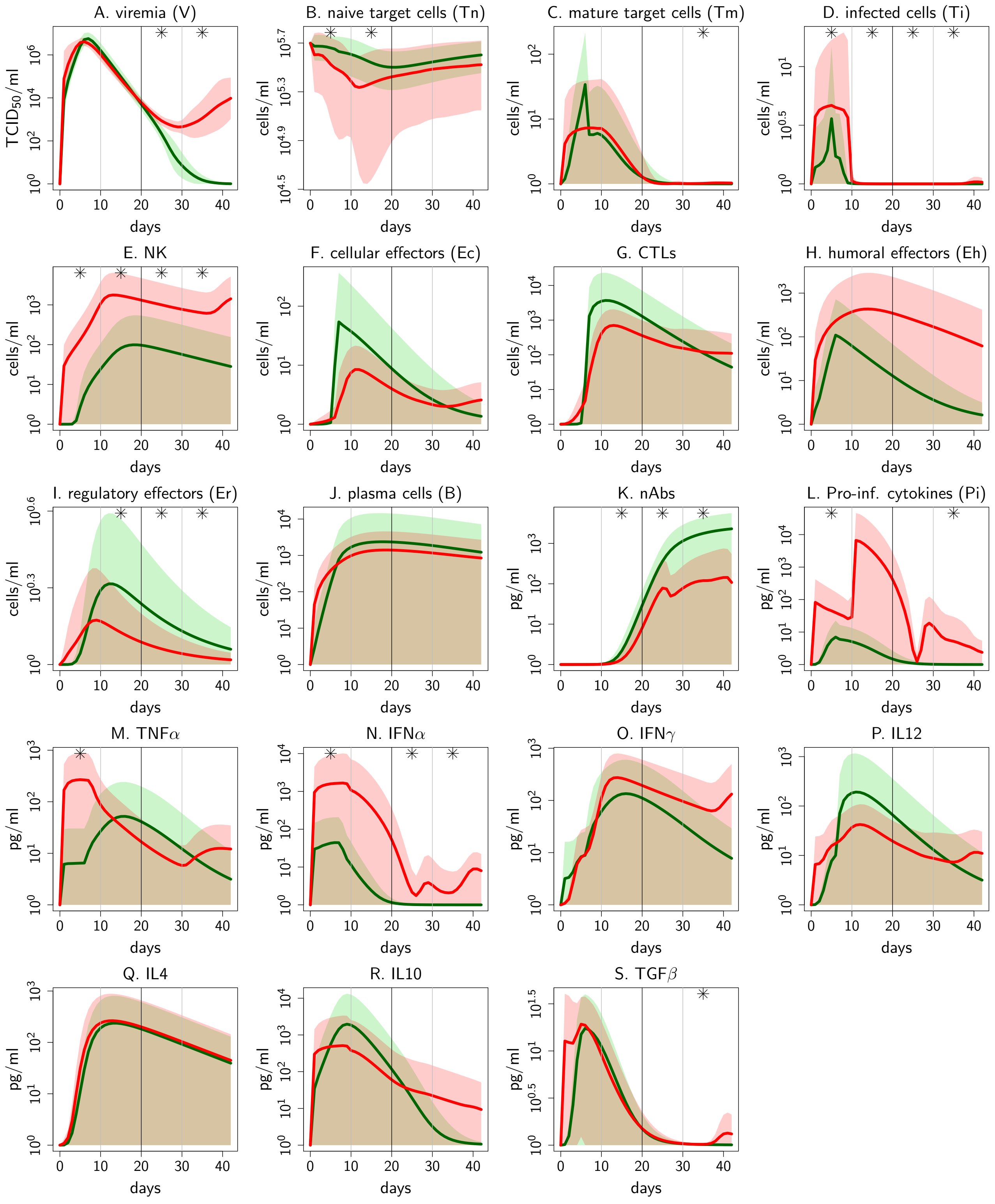

Supplement: Supplementary file 1 — Within-host dynamics. Evolution over infection time of the 19 state variables of the model: free viral particles, target cells (APC), natural killers, adaptive effectors including cytotoxic T lymphocytes, plasma cells, neutralising antibodies and cytokines. Comparison between uniphasic and biphasic viremia profiles. (PDF 124 kb) [file 12918_2018_666_MOESM1_ESM.pdf]
