# Supplementary material for: How to prevent viremia rebound? Evidence from a PRRSv data-supported model of immune response
Source: BMC Syst Biol. 2019 Jan 29;13:15. doi: 10.1186/s12918-018-0666-7 (PMC6352383; doi:10.1186/s12918-018-0666-7)

The figure provides values of the 14 parameters linked to between-host variability: **A-B** host–virus interactions; **C** viral replication; **D-F** activation of the adaptive response; **G-N** cytokine syntheses by activated target cells (except L: by humoral effectors). Rate values are presented in relative scale, *i.e.* normalised according to their assumed upper and lower boundaries (see **Table A5-4** in **Additional file 5 – Model description & Sensitivity analyses**. Mean value and standard deviation of the 35 representative individuals selected for the uniphasic (green) and biphasic (red) viremia profiles over the parameter ranges.
$*$ p-value$<$ 0.01 when comparing uniphasic and biphasic profiles (Kolmogorov–Smirnov test).

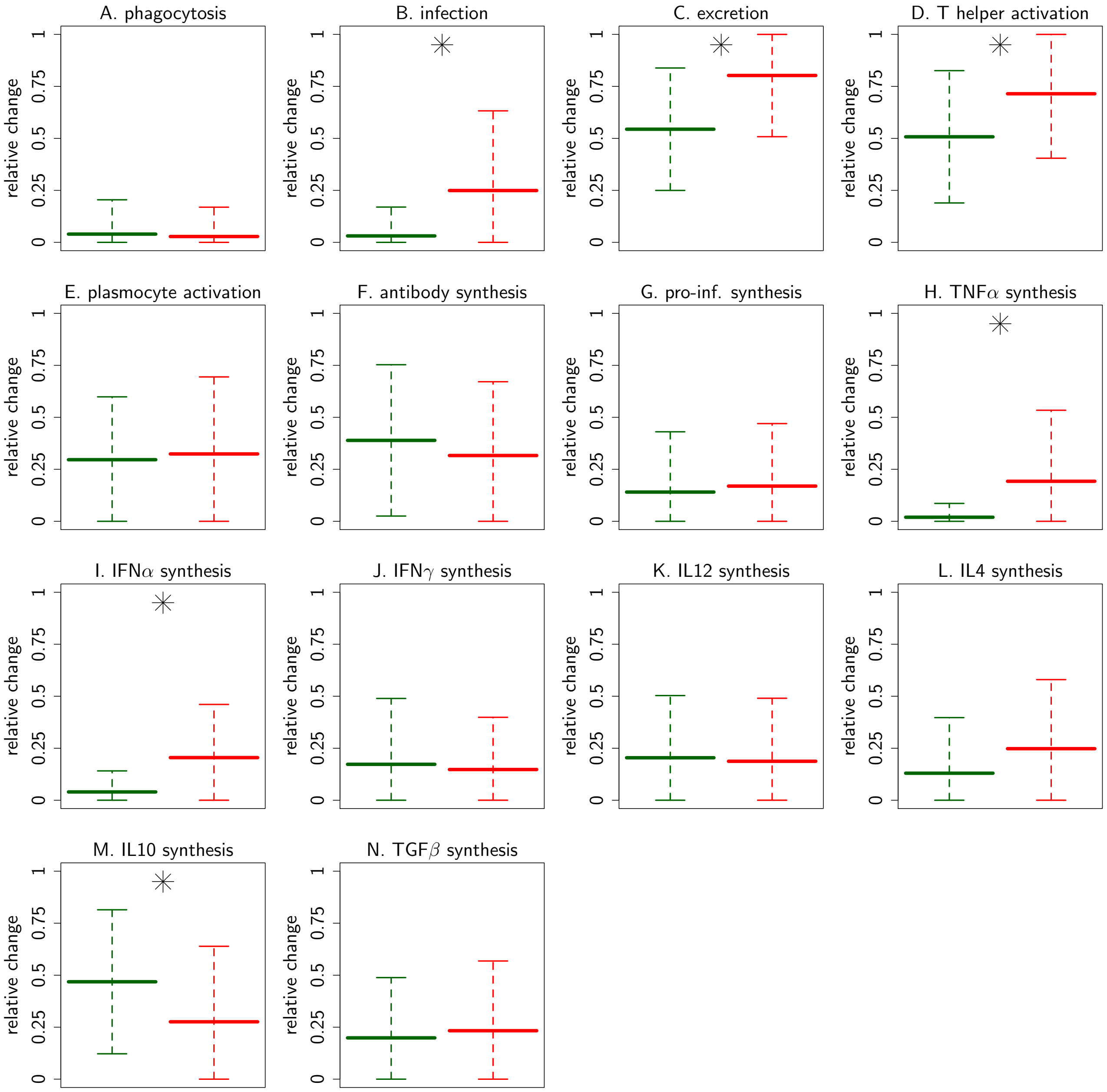

Supplement: Supplementary file 2 — Estimated parameters. Values of the 14 parameters linked to between-host variability: host–virus interactions, viral replication, activation of the adaptive response, cytokine syntheses by activated target cells; for the 35 representative individuals selected for the uniphasic and biphasic viremia profiles. Comparison between uniphasic and biphasic viremia profiles. (PDF 87 kb) [file 12918_2018_666_MOESM2_ESM.pdf]
