# Supplementary material for: How to prevent viremia rebound? Evidence from a PRRSv data-supported model of immune response
Source: BMC Syst Biol. 2019 Jan 29;13:15. doi: 10.1186/s12918-018-0666-7 (PMC6352383; doi:10.1186/s12918-018-0666-7)
Supplement: Supplementary file 3 — Mechanisms resulting in viremia profile inversion. Percentage of individuals, among the 35 representative individuals selected for the uniphasic and biphasic viremia profiles, that had a profile inversion when boosting or inhibiting (depending on the profile) either mechanism (one at a time): infection, apoptosis by TNFα, cytolysis by cytotoxic T lymphocytes, neutralisation by antibodies. Cytolysis by natural killers never resulted in a profile inversion (not shown). (PDF 87 kb) [file 12918_2018_666_MOESM3_ESM.pdf]

The table provides percentage of individuals, among the 35 representative individuals selected for **A:** the uniphasic (green) and **B:** biphasic (red) viremia profiles, that had a profile inversion (PI) when boosting or inhibiting (depending on the profile) either mechanism (one at a time): infection (Inf), apoptosis by (Ap), cytolysis by cytotoxic T lymphocytes (CyLc), neutralisation by antibodies (Neutr). Cytolysis by natural killers never resulted in a profile inversion (not shown).
Last rows in both tables: percentage for each mechanism (first 4 columns), or for at least one mechanism (last column). Other rows: percentage (last column) for and only for the mechanisms tagged by a • (first 4 columns). *For instance, all 35 individuals of the biphasic viremia profile had a profile inversion; 80% of these individuals changed to a uniphasic viremia profile when inhibiting apoptosis, out of which 54.3% of all 35 individuals also changed when boosting cytolysis or neutralisation, but not when inhibiting infection.*

A. uniphasic

Inf	Ap	CyLc	Neutr	% of PI
●				6
	●			26
		●		6
			●	0
●	●			9
●		●		9
●			●	0
	●	●		23
	●		●	0
		●	●	0
●	●	●		0
●	●		●	0
●		●	●	0
	●	●	●	0
●	●	●	●	0
% of PI 23	57	37	0	77

B. biphasic

Inf	Ap	CyLc	Neutr	% of PI
●				0
	●			0
		●		3
			●	0
●	●			0
●		●		0
●			●	0
	●	●		6
	●		●	0
		●	●	14
●	●	●		0
●	●		●	0
●		●	●	3
	●	●	●	54
●	●	●	●	20
% of PI 23	80	100	91	100
