# Supplementary material for: How to prevent viremia rebound? Evidence from a PRRSv data-supported model of immune response
Source: BMC Syst Biol. 2019 Jan 29;13:15. doi: 10.1186/s12918-018-0666-7 (PMC6352383; doi:10.1186/s12918-018-0666-7)
Supplement: Supplementary file 4 — Smoothed viremia data. Parameters of the Wood’s functions fitted to the PHGC data [19], for the selected individuals exhibiting uniphasic or biphasic profiles. (PDF 121 kb) [file 12918_2018_666_MOESM4_ESM.pdf]

The table provides the parameters of the Wood's functions fitted to the PHGC data [Islam *et al.* 2013], for the selected individuals exhibiting uniphasic (UNI) or biphasic (BI) profiles. Equations of the Wood's functions, defining viremia V (in TCID_{50}/ml) for time $t \in [0, 42]$ days post infection, are:

$$\log\big(V(t)\big) = \begin{cases} A1\, t^{B1}\ \exp^{-C1\, t} & \text{Uniphasic} \\ A1\, t^{B1}\ \exp^{-C1\, t} & \text{Biphasic \& } t \leqslant Dt \\ A1\, t^{B1}\ \exp^{-C1\, t} + \max\big(0, A2\,(t-Dt)^{B2}\ \exp^{-C2\,(t-Dt)}\big) & \text{Biphasic \& } t > Dt \end{cases}$$

Profile	A1	B1	C1
Uniphasic	1.868	1.269	0.156
Uniphasic	2.962	1.024	0.157
Uniphasic	1.179	1.511	0.167
Uniphasic	4.134	0.664	0.111
Uniphasic	2.765	1.044	0.147
Uniphasic	3.126	0.833	0.118
Uniphasic	3.726	0.837	0.134
Uniphasic	3.331	0.883	0.133
Uniphasic	4.226	0.734	0.123
Uniphasic	2.945	0.947	0.139
Uniphasic	3.152	0.951	0.145
Uniphasic	2.451	1.082	0.144
Uniphasic	2.498	1.161	0.166
Uniphasic	2.299	1.211	0.17
Uniphasic	2.479	1.135	0.159
Uniphasic	1.606	1.286	0.146
Uniphasic	1.877	1.208	0.143
Uniphasic	2.721	1.087	0.16
Uniphasic	2.611	1.009	0.141
Uniphasic	2.296	1.217	0.182
Uniphasic	3.255	0.926	0.141
Uniphasic	2.159	1.163	0.152
Uniphasic	3.108	0.928	0.132
Uniphasic	3.354	0.957	0.15
Uniphasic	3.667	0.791	0.128
Uniphasic	2.689	1.102	0.158
Uniphasic	2.579	1.049	0.141
Uniphasic	2.104	1.203	0.153
Uniphasic	2.956	0.886	0.128
Uniphasic	2.554	1.128	0.158
Uniphasic	2.318	1.068	0.134
Uniphasic	2.762	1.004	0.137
Uniphasic	3.678	0.848	0.136
Uniphasic	3.965	0.701	0.113
Uniphasic	2.834	1.06	0.159
Uniphasic	2.276	1.176	0.157
Uniphasic	3.183	0.848	0.122
Uniphasic	2.628	1.086	0.159
Uniphasic	2.914	1.108	0.171
Uniphasic	2.156	1.252	0.174
Uniphasic	2.574	1.023	0.136
Uniphasic	1.961	1.196	0.145
Uniphasic	2.933	1.089	0.171
Uniphasic	4.458	0.691	0.122
Uniphasic	2.377	1.07	0.135
Uniphasic	1.462	1.386	0.16
Uniphasic	3.204	0.998	0.157

Profile	A1	B1	C1	A2	B2	C2	Dt
Biphasic	1.12	1.681	0.212	6.399	8.918	3.343	32
Biphasic	2.545	1.033	0.142	6.047	7.606	2.876	32
Biphasic	1.262	1.481	0.174	4.391	4.768	1.807	33
Biphasic	2.128	1.192	0.162	9.48	6.924	2.989	33
Biphasic	2.084	1.114	0.147	1.596	8.339	2.88	32
Biphasic	2.791	0.976	0.142	9.937	4.726	1.984	32
Biphasic	1.656	1.353	0.167	6.429	7.379	2.856	32
Biphasic	1.125	1.474	0.162	0.798	6.797	1.952	31
Biphasic	1.913	1.119	0.131	3.336	6.472	2.417	25
Biphasic	2.073	1.046	0.121	7.442	2.657	1.367	33
Biphasic	1.251	1.496	0.178	4.283	2.469	0.798	30
Biphasic	1.941	1.083	0.127	3.547	5.916	2.196	32
Biphasic	1.853	1.215	0.151	6.857	8.487	3.361	26
Biphasic	1.625	1.311	0.167	6.033	4.585	1.781	33
Biphasic	2.448	0.985	0.128	5.674	5.891	2.226	32
Biphasic	2.069	1.166	0.148	7.36	3.083	1.238	33
Biphasic	1.997	1.276	0.178	8.755	8.198	3.13	32
Biphasic	1.397	1.527	0.205	3.768	9.351	3.351	32
Biphasic	1.484	1.393	0.169	6.451	9.145	3.389	33
Biphasic	2.199	1.184	0.168	7.633	8.565	3.328	25
Biphasic	2.503	0.933	0.119	8.669	6.844	2.825	32
Biphasic	1.187	1.475	0.167	1.667	5.576	1.712	30
Biphasic	2.136	1.019	0.117	8.625	5.759	2.434	33
Biphasic	2.897	0.891	0.12	6.159	9.552	3.661	26
Biphasic	1.337	1.477	0.175	8.537	2.1	0.917	31
Biphasic	1.634	1.472	0.205	9.24	4.331	1.732	32
Biphasic	2.524	1.031	0.144	5.54	9.033	3.566	32
Biphasic	1.892	1.268	0.165	0.178	5.929	1.268	29
Biphasic	2.648	1.017	0.147	9.806	6.664	2.791	26
Biphasic	1.886	1.264	0.167	7.445	3.843	1.672	33
Biphasic	2.643	0.959	0.137	4.785	2.686	1.084	32
Biphasic	2.054	1.337	0.192	7.337	3.414	1.44	26
Biphasic	2.68	0.987	0.142	8.383	9.38	3.738	33
Biphasic	2.664	0.931	0.129	5.95	9.841	3.72	32
Biphasic	3.817	0.712	0.117	7.914	3.063	1.483	26
Biphasic	3.577	0.737	0.113	4.562	2.345	0.972	40
Biphasic	3.536	0.801	0.128	5.525	9.018	3.434	33
Biphasic	2.954	0.856	0.121	1.16	9.425	3.094	32
Biphasic	1.574	1.315	0.157	4.132	2.861	1.151	33
Biphasic	3.713	0.817	0.143	8.06	3.112	1.357	32
Biphasic	3.277	0.892	0.136	2.403	8.543	2.937	38
Biphasic	2.068	1.156	0.144	0.612	2.539	0.457	33
Biphasic	2.796	0.988	0.152	6.748	4.37	1.83	33
Biphasic	2.444	1.201	0.183	3.348	5.793	1.888	37
Biphasic	1.894	1.147	0.139	9.508	3.457	1.683	33
Biphasic	2.04	1.089	0.138	2.247	9.535	3.321	32
Biphasic	2.174	1.037	0.131	9.495	4.952	2.202	32

A4 table – *Continued to next page*

A4 table– *Continued from previous page*

Profile	A1	B1	C1
Uniphasic	2.486	1.09	0.154
Uniphasic	1.844	1.172	0.138
Uniphasic	3.329	0.926	0.144
Uniphasic	4.793	0.603	0.106
Uniphasic	2.692	0.955	0.131
Uniphasic	2.851	0.95	0.128
Uniphasic	1.695	1.437	0.191
Uniphasic	2.833	1.02	0.152
Uniphasic	2.133	1.341	0.192
Uniphasic	3.296	0.972	0.152
Uniphasic	3.736	0.814	0.131
Uniphasic	3.51	0.792	0.117
Uniphasic	2.489	1.013	0.13
Uniphasic	3.491	0.771	0.117
Uniphasic	3.762	0.815	0.145
Uniphasic	3.064	0.858	0.122
Uniphasic	2.917	1.004	0.144
Uniphasic	4.546	0.646	0.109
Uniphasic	3.025	0.922	0.129
Uniphasic	3.869	0.743	0.115
Uniphasic	2.031	1.183	0.143
Uniphasic	2.121	1.146	0.139
Uniphasic	3.398	0.823	0.131
Uniphasic	3.784	0.775	0.132
Uniphasic	4.067	0.678	0.113
Uniphasic	3.715	0.756	0.124
Uniphasic	3.938	0.74	0.122
Uniphasic	2.117	1.104	0.138
Uniphasic	2.548	0.984	0.128
Uniphasic	2.214	1.043	0.131
Uniphasic	2.352	1.12	0.152
Uniphasic	3.631	0.76	0.121
Uniphasic	3.899	0.733	0.12
Uniphasic	4.052	0.702	0.122
Uniphasic	2.29	1.091	0.145
Uniphasic	2.496	1.038	0.132
Uniphasic	3.105	0.884	0.126
Uniphasic	3.343	0.872	0.128
Uniphasic	1.726	1.277	0.15
Uniphasic	2.904	0.941	0.134
Uniphasic	2.267	1.035	0.136
Uniphasic	2.609	1.085	0.154
Uniphasic	1.713	1.199	0.144
Uniphasic	3.052	0.946	0.135
Uniphasic	2.468	1.011	0.129
Uniphasic	4.072	0.75	0.124
Uniphasic	2.719	0.947	0.132
Uniphasic	2.468	1.089	0.15
Uniphasic	2.486	1.073	0.146
Uniphasic	3.104	0.933	0.135
Uniphasic	2.114	1.234	0.162
Uniphasic	1.93	1.171	0.139
Uniphasic	4.043	0.692	0.116
Uniphasic	1.269	1.568	0.184
Uniphasic	1.777	1.268	0.157

Profile	A1	B1	C1	A2	B2	C2	Dt
Biphasic	3.094	0.867	0.135	5.635	1.277	0.63	20
Biphasic	1.551	1.359	0.172	8.9	7.199	2.966	25
Biphasic	4.45	0.61	0.108	1.362	7.769	2.437	32
Biphasic	1.498	1.426	0.183	5.835	6.263	2.273	31
Biphasic	2.079	1.081	0.123	4.139	6.914	2.555	33
Biphasic	1.615	1.533	0.208	6.149	6.737	2.57	32
Biphasic	4.502	0.579	0.098	9.259	6.208	2.638	26
Biphasic	2.503	1.012	0.137	4.842	4.913	1.956	25
Biphasic	2.048	1.256	0.172	5.14	2.84	1.177	25
Biphasic	3.356	0.762	0.117	7.069	4.384	1.894	33
Biphasic	2.847	1.087	0.175	5.812	5.549	2.12	33
Biphasic	3.72	0.759	0.118	8.127	2.249	1.102	25
Biphasic	2.425	1.122	0.157	4.481	8.388	3.033	24
Biphasic	2.875	0.952	0.132	3.385	5.542	1.853	31
Biphasic	2.596	0.962	0.119	6.707	2.003	1.075	33
Biphasic	2.181	1.115	0.147	7.541	6.61	2.679	25
Biphasic	2.335	1.102	0.151	4.805	0.449	0.368	26
Biphasic	1.24	1.529	0.177	6.051	3.27	1.324	26
Biphasic	1.883	1.208	0.144	3.635	8.679	3.18	32
Biphasic	3.139	0.837	0.111	0.247	9.498	2.666	25
Biphasic	2.223	1.175	0.158	8.668	3.136	1.529	25
Biphasic	2.051	1.108	0.132	6.618	4.496	1.751	37
Biphasic	2.003	1.32	0.184	5.838	7.448	2.965	25
Biphasic	2.154	1.088	0.134	8.885	6.49	2.724	32
Biphasic	3.103	0.866	0.12	3.302	9.264	3.288	33
Biphasic	2.407	1.07	0.14	6.98	7.963	2.977	31
Biphasic	2.713	0.903	0.116	9.961	9.851	4.029	25
Biphasic	2.251	1.114	0.144	3.797	1.162	0.386	30
Biphasic	1.643	1.302	0.163	9.161	0.264	1.011	34
Biphasic	1.862	1.308	0.171	9.793	6.226	2.671	32
Biphasic	2.034	1.113	0.132	4.311	7.235	2.702	26
Biphasic	3.65	0.683	0.103	2.715	6.681	2.189	38
Biphasic	1.579	1.416	0.172	7.926	6.935	2.683	32
Biphasic	2.047	1.152	0.147	5.766	0.319	0.741	34
Biphasic	1.683	1.463	0.194	5.284	5.92	2.356	25
Biphasic	1.647	1.281	0.146	8.87	5.8	2.528	33
Biphasic	1.186	1.663	0.204	4.692	3.289	1.29	31
Biphasic	2.475	0.968	0.121	8.997	8.089	3.302	37
Biphasic	3.149	0.846	0.122	7.607	5.497	2.353	26
Biphasic	2.13	1.119	0.142	9.591	5.199	2.252	26
Biphasic	2.236	1.026	0.129	6.335	5.845	2.278	33
Biphasic	2.963	0.841	0.12	6.933	8.649	3.259	33
Biphasic	1.79	1.12	0.129	3.3	7.007	2.544	37
Biphasic	2.133	1.089	0.14	7.293	3.863	1.59	32
Biphasic	2.913	0.897	0.13	1.56	3.003	0.801	34
Biphasic	1.505	1.256	0.141	3.811	0.701	0.147	39
Biphasic	1.993	1.298	0.179	9.355	6.416	2.601	32
Biphasic	2.459	1.171	0.184	9.627	6.11	2.457	25
Biphasic	1.549	1.335	0.154	5.679	4.573	1.814	39
Biphasic	1.905	1.087	0.128	2.522	7.073	2.425	32
Biphasic	2.477	1.102	0.154	3.12	4.698	1.585	26
Biphasic	1.787	1.178	0.138	5.6	0.828	0.484	32
Biphasic	1.701	1.239	0.149	6.965	2.358	1.002	32
Biphasic	1.437	1.386	0.17	5.516	8.939	3.392	25
Biphasic	1.196	1.611	0.204	7.878	9.426	3.7	33

A4 table – *Continued to next page*

A4 table– *Continued from previous page*

Profile	A1	B1	C1
Uniphasic	1.92	1.366	0.186
Uniphasic	2.319	1.001	0.126
Uniphasic	3.515	0.741	0.118
Uniphasic	2.517	0.995	0.131
Uniphasic	2.651	0.965	0.129
Uniphasic	1.744	1.274	0.155
Uniphasic	2.326	1.087	0.144
Uniphasic	2.229	1.068	0.135
Uniphasic	1.957	1.192	0.154
Uniphasic	1.767	1.254	0.148
Uniphasic	1.973	1.169	0.142
Uniphasic	2.566	1.079	0.148
Uniphasic	3.618	0.772	0.127
Uniphasic	2.305	1.159	0.154
Uniphasic	2.979	0.965	0.135
Uniphasic	1.75	1.267	0.148
Uniphasic	2.389	1.134	0.156
Uniphasic	2.418	1.064	0.136
Uniphasic	2.49	1.102	0.152
Uniphasic	3.201	0.814	0.12
Uniphasic	3.579	0.772	0.117
Uniphasic	2.463	1.129	0.159
Uniphasic	2.698	1.084	0.162
Uniphasic	3.783	0.775	0.118
Uniphasic	3.314	0.885	0.135
Uniphasic	2.774	1.018	0.144
Uniphasic	3.375	0.761	0.113
Uniphasic	2.874	0.916	0.127
Uniphasic	4.624	0.636	0.11

Profile	A1	B1	C1	A2	B2	C2	Dt
Biphasic	2.462	1.012	0.133	5.185	4.694	1.899	25
Biphasic	2.221	1.017	0.123	8.21	0.805	0.683	33
Biphasic	1.489	1.345	0.162	9.109	6.214	2.598	26
Biphasic	3.091	0.862	0.128	0.432	8.321	2.365	24
Biphasic	1.397	1.353	0.152	7.211	4.789	2.028	33
Biphasic	2.644	1.029	0.142	9.17	6.304	2.464	31
Biphasic	1.888	1.181	0.146	6.357	0.22	0.229	32
