# Supplementary material for: How to prevent viremia rebound? Evidence from a PRRSv data-supported model of immune response
Source: BMC Syst Biol. 2019 Jan 29;13:15. doi: 10.1186/s12918-018-0666-7 (PMC6352383; doi:10.1186/s12918-018-0666-7)
Supplement: Supplementary file 5 — Model description & Sensitivity analyses. The file provides a complete description of the dynamic model representing the within-host dynamics induced by a primary PRRSv infection in a naive pig. It specifies the modelling assumptions and includes all model equations. The file also describes the global sensitivity analyses performed to assess the impact of model parameters on the viral dynamics. Corresponding aims, methods and results are presented. (PDF 710 kb) [file 12918_2018_666_MOESM5_ESM.pdf]

How to Prevent Viremia Rebound? Evidence from a PRRSv Data-Supported Model of Immune Response

Additional file 5

Model description & Sensitivity analyses

Natacha Go[1,2,3*], Zeenath Islam[3], Catherine Belloc[1], Suzanne Touzeau[2,4,†], Andrea Doeschl-Wilson[3,†]

1 BIOEPAR, INRA, Oniris, Nantes, France
2 BIOCORE, Inria, INRA, CNRS, UPMC Univ Paris 06, Université Côte d'Azur, France
3 Division of Genetics and Genomics, The Roslin Institute, Midlothian, UK
4 ISA, INRA, CNRS, Université Côte d'Azur, France

†These authors contributed equally to this work.

* natacha.go@protonmail.com

1 Model description

The model simulates the infection and immune dynamics induced by a primary PRRSv infection in a PRRSv-naive post-weaning pig. It represents the mechanisms at the between-cell scale and provides an integrative view of the immune response. It extends a previous model [1] to get a mechanistic view of the whole immune response, by detailing the activation and orientation steps of the adaptive response.

The resulting model is a deterministic dynamic model in continuous time, formalised by a set of ordinary differential equations. They describe the evolution of the concentration of 19 state variables, consisting of the viral particles, the main innate and adaptive immune cells and the major cytokines. The state variables are presented in Table A5-1. Their interactions are illustrated in Figure 9 in the main text. Their dynamics are described below: first the viral particles (section 1.1) and their target cells, the antigen-presenting cells (APC) (section 1.2); then the innate (section 1.3) and adaptive effectors (section 1.4), followed by the neutralising antibodies (section 1.5); and finally the cytokines (section 1.6). Most immune processes are regulated by a complex cytokine feedback system: activated immune cells synthesise cytokines, which up- and/or down-regulate the cell functions (section 1.6.1). All parameters are listed in Table A5-4.

1.1 Free viral particles

Exposure The virus enters the body through the mucosal surfaces of the respiratory tract. The viral particles then migrate to the lungs, their main infection place. Meanwhile, they face the first line of defence of the organism (physical barriers and epithelial cells), which slow down their progression.

In the data, pigs are experimentally infected by a fixed inoculum. The simplest way to represent such an exposure is a positive initial condition for the free viral particles. However, the model represents the within-host dynamics in the infection place, not the host first line of defence. So we assumed that the inoculation corresponds to a gradual input of viral particles in the lung, that follows a narrow bell-shaped curve [2, chap. 3] defined as:

$$E(t) = \begin{cases} \mathcal{N}_E \, \frac{t^{a_E-1}\,(D_E-t)^{b_E-1}}{\int_0^{D_E} t^{a_E-1}\,(D_E-t)^{b_E-1}\,dt} & \text{if } 0 < t < D_E \\ 0 & \text{else} \end{cases}$$

with

$a_E, b_E > 1$	shape parameters (no unit),
$\mathcal{N}_E$	exposure intensity (in TCID_{50}/ml),
D_E	exposure duration (in days).

$a_E = b_E$ produces a symmetric bell-shaped curve, $a_E < b_E$ a left-shifted bell-shaped curve.

The exposure intensity or total viral dose ($\mathcal{N}_E$) corresponds to the experimental inoculum dose. The parameter values calibrated on the PHGC data are presented in Table A5-4.

Viral dynamics When free viral particles (V) encounter naive target cells, they can either be phagocyted (rate η_T), resulting in viral destruction and cell activation, or they can infect the cells (rate β_T), resulting in virus replication and release of new particles (rate e_T). The replication is inhibited by antiviral cytokines TNFα, IFNα, IFNγ. Free viral particles are subject to natural decay and migration (rate μ_V^{nat}). They can also be neutralised by antibodies nAb (rate μ_V^{ad}).

Table A5-1. State variables of the model.

Var.	Name	Description	Eq.
PRRS virus			
V	free Viral particles	Circulating viral particles, initiated by the viral exposure. Can infect or be phagocyted by target cells, or neutralised by antibodies.	(A5-1)
Target cells: antigen-presenting cells (APC)			
T_n	Naive Target cells	Naive and permissive APC (have never encountered the virus); group the naive macrophages, conventional and plasmacytoid dendritic cells. Can phagocyte the virus or become infected.	(A5-2)
T_m	Mature Target cells	Activated, permissive and non infected APC; group the phagocyting macrophages, mature conventional and plasmacytoid dendritic cells. Can phagocyte the virus or become infected; synthesise cytokines and activate the adaptive response.	(A5-3)
T_i	Infected Target cells	Activated, non permissive and infected APC; group the infected macrophages and mature conventional dendritic cells. Synthesise cytokines and activate the adaptive response.	(A5-4)
Activated innate effector			
NK	Natural Killers	Cytolyse infected cell cytolysis and synthesise antiviral cytokines.	(A5-5)
Activated adaptive effectors: PRRSv-specific T cells			
CTL	Cytotoxic T Lymphocytes	CD_8^+ T lymphocytes. Cytolyse the infected cells and synthesise antiviral cytokines.	(A5-9)
E_c	Cellular Effectors	Type 1 helper T cells (T_{h1}), a CD_4^+ T lymphocyte subtype. Activate CTL and IgG-producing plasma cells, synthesise antiviral cytokine.	(A5-6)
E_h	Humoral Effectors	Type 2 helper T cells(T_{h2}), a CD_4^+ T lymphocyte subtype. Activate IgM-producing plasma cells and synthesise immuno-modulatory cytokines.	(A5-7)
E_r	Regulatory Effectors	Regulatory T cells (T_{reg}) and Type 17 helper T cells (Th_{17}): CD_4^+ T lymphocyte subtypes. Activate IgA-producing plasma cells and synthesise immuno-modulatory cytokines.	(A5-8)
Neutralising antibodies			
B	plasma cells	Activated IgG-, IgM- and IgA-producing plasma cells.	(A5-10)
nAb	Neutralising Antibodies	IgG, IgM and IgA neutralising antibodies.	(A5-11)
Cytokines			
Pi	Pro-Inflammatory	Group IL1β, IL6, IL8 and CCL2. Amplify the recruitment of APC and NK on the infection place; IL6 inhibits the differentiation of helper T cells towards T_{h1} and T_{reg}; IL1β induces the synthesis of IL6 and IL8.	(A5-12)
TNFα	Tumour Necrosis Factor α	Innate antiviral and pro-inflammatory cytokine. Induces the apoptosis of APC and T cells.	(A5-13)
IFNα	Type 1 interferon	Innate antiviral cytokine.	(A5-14)
IFNγ	Type 2 interferon	Adaptive antiviral and immuno-regulatory cytokine. Induces the differentiation of helper T cells towards T_{h1}; auto-amplifies its synthesis.	(A5-15)
IL12	Interleukin 12	Immuno-regulatory cytokine. Amplifies the recruitment of APC and NK on the infection place, the activation of NK and the proliferation of T cells; induces the differentiation of helper T cells towards T_{h1}.	(A5-18)
IL4	Interleukin 4	Immuno-regulatory cytokine. Amplifies the differentiation of helper T cells towards T_{h2}.	(A5-19)
IL10	Interleukin 10	Immuno-modulatory cytokine. Inhibits the activation of NK and the synthesis of IL1β, TNFα, IL12 and IFNγ; auto-amplifies its synthesis by E_h.	(A5-16)
TGFβ	Transforming Growth Factor β	Immuno-modulatory cytokine. Inhibits the proliferation of lymphocytes and their immune functions.	(A5-17)

Free viral particles:

$$
\begin{aligned}
\dot{V} = &+ E(t) && \longleftarrow \text{exposure} \\
&- u_T^V\, \eta_T\, V\, (T_n + T_m)\, \kappa^-(\text{IL10} + \text{TGF}\beta)\, [1 + \kappa^+(\text{TNF}\alpha + \text{IFN}\alpha + \text{IFN}\gamma)] && \longleftarrow \text{phagocytosis} \\
&- u_T^V\, \beta_T\, V\, (T_m + T_n)\, \kappa^-(\text{TNF}\alpha + \text{IFN}\alpha + \text{TGF}\beta)\, [1 + \kappa^+(\text{IL10})] && \longleftarrow \text{infection} \qquad \text{(A5-1)} \\
&+ e_T\, T_i\, \kappa^-(\text{TNF}\alpha + \text{IFN}\alpha + \text{IFN}\gamma) && \longleftarrow \text{excretion} \\
&- V\, \left(\mu_V^{\text{nat}} + \mu_V^{\text{ad}}\, \text{nAb}\right) && \longleftarrow \text{decay \& neutralisation}
\end{aligned}
$$

1.2 Antigen-presenting cells

PRRSv replicates in antigen-presenting cells (APC), primarily in pulmonary macrophages and secondarily in dendritic cells [3]. Conventional and plasmacytoid dendritic cells are the two major subtypes involved in PRRSv infection [4, 5]. They all have similar key immune functions, that are expressed once activated by the binding of a free viral particle.

- Macrophages are responsible for the phagocytosis of viral particles; the synthesis of pro-inflammatory (Pi : IL1β, IL6, IL8, CCL2), antiviral (IFNα, TNFα) and immuno-regulatory (IL12, IL10, TGFβ) cytokines; and [6]; and to a lesser extent, the adaptive response activation. They are permissive to PRRSv.
- Conventional dendritic cells are responsible for various cytokine syntheses (IL1β, IL6, IL8, IFNα, TNFα, IL12, IFNγ, IL10 and TGFβ), the adaptive response activation and, to a lesser extent, the phagocytosis of viral particles. Their permissiveness to PRRSv is lower than the macrophage permissiveness [7], but they show similar PRRSv replication rates once infected [7–9].
- Plasmacytoid dendritic cells are responsible for the synthesis of innate antiviral cytokines (IFNα and TNFα) and, to a lesser extent, the phagocytosis of viral particles. They do not allow PRRSv replication [7, 8, 10, 11].

PRRSv hampers APC immune functions in a similar way [12], so they have very similar dynamics and impacts throughout PRRSv infection [8, 10]. Therefore, we grouped these three cell types in the model into a *target cell* functional group. We defined three target cell states: naive (T_n), *i.e.* which has never encountered the virus; mature (T_m), *i.e.* which has been activated by phagocyting the virus; and infected (T_i), *i.e.* which has been activated and releases new viral particles.

Recruitment Naive target cells are recruited from the bloodstream (constant rate R_T). Cytokines IL6 and IL12 co-amplify their recruitment in synergy and IL8 and CCL2 attracts the naive target cells to the infection place [6, 13–15]. As we grouped pro-inflammatory cytokines in the model into Pi, recruitment is co-amplified by Pi and IL12.

Decay All target cell states are subject to natural decay and/or migration (rate μ_T^{nat}), as well as apoptosis induced by TNFα (rate μ_T^{ap}) [12, 16]. The natural decay rate is considered higher for infected cells than for naive and mature target cells (multiplicative factor δ_μ). Moreover, infected target cells can be destroyed by natural killers (rate μ_T^{inn}) and cytotoxic T cells (rate μ_T^{ad}).

State changes When naive target cells encounter free viral particles (V), they can either phagocyte the virus (rate η_T) or become infected (rate β_T). We assumed that phagocyting target cells revert to the naive state after activation loss (rate γ_T), whereas infected target cells remain infected (*i.e.* they cannot eliminate the virus). As the phagocytosis of a viral particle lasts between 1 and 4 hours, we neglected this duration. We also assumed that cells activated by phagocytosis can be infected. However, once infected, target cells cannot be infected by other viral particles [17].

Cytokines are involved in the regulation of these mechanisms [18–21]. Phagocytosis is amplified by the antiviral cytokines (TNFα, IFNα, IFNγ) and inhibited by the immuno-modulatory cytokines (IL10, TGFβ). Activation loss is amplified by the immuno-modulatory cytokines. Infection is amplified by IL10 and inhibited by innate antiviral cytokines (TNFα, IFNα) and TGFβ.

Naive target cells:

$$
\begin{aligned}
\dot{T_n} = &+ R_T\, [1 + \kappa^+(\text{IL12}\,\text{Pi})] && \longleftarrow \text{recruitment} \\
&- \eta_T\, T_n\, V\, \kappa^-(\text{IL10} + \text{TGF}\beta)\, [1 + \kappa^+(\text{TNF}\alpha + \text{IFN}\alpha + \text{IFN}\gamma)] && \longleftarrow \text{activation} \\
&+ \gamma_T\, T_m\, [1 + \kappa^+(\text{IL10} + \text{TGF}\beta)] && \longleftarrow \text{activation loss} \qquad \text{(A5-2)} \\
&- \beta_T\, T_n\, V\, \kappa^-(\text{TNF}\alpha + \text{IFN}\alpha + \text{TGF}\beta)\, [1 + \kappa^+(\text{IL10})] && \longleftarrow \text{infection} \\
&- T_n\, \left(\mu_T^{\text{nat}} + \mu_T^{\text{ap}}\, \kappa^+(\text{TNF}\alpha)\right) && \longleftarrow \text{decay \& apoptosis}
\end{aligned}
$$

Mature target cells:

$$\begin{aligned}
\dot{T_m} = &+ \eta_T\, T_n\, V\, \kappa^-(\text{IL10} + \text{TGF}\beta)\, [1 + \kappa^+(\text{TNF}\alpha + \text{IFN}\alpha + \text{IFN}\gamma)] && \longleftarrow \text{activation} \\
&- \gamma_T\, T_m\, [1 + \kappa^+(\text{IL10} + \text{TGF}\beta)] && \longleftarrow \text{activation loss} \\
&- \beta_T\, T_m\, V\, \kappa^-(\text{TNF}\alpha + \text{IFN}\alpha + \text{TGF}\beta)\, [1 + \kappa^+(\text{IL10})] && \longleftarrow \text{infection} \\
&- T_m\, \left(\mu_T^{\text{nat}} + \mu_T^{\text{ap}}\, \kappa^+(\text{TNF}\alpha)\right) && \longleftarrow \text{decay \& apoptosis}
\end{aligned} \tag{A5-3}$$

Infected target cells:

$$\begin{aligned}
\dot{T_i} = &+ \beta_T\, (T_m + T_n)\, V\, \kappa^-(\text{TNF}\alpha + \text{IFN}\alpha + \text{TGF}\beta)\, [1 + \kappa^+(\text{IL10})] && \longleftarrow \text{infection} \\
&- T_i\, \left(\mu_T^{\text{nat}}\, \delta_\mu + \mu_T^{\text{ap}}\, \kappa^+(\text{TNF}\alpha) + (\mu_T^{\text{inn}}\, \text{NK} + \mu_T^{\text{ad}}\, \text{CTL})\right) && \longleftarrow \text{decay \& apoptosis}
\end{aligned} \tag{A5-4}$$

N.B. Without infection, the cytokine concentrations are supposed to be negligible and the resulting concentration of naive target cells is constant: $T_n = \frac{R_T}{\mu_T^{\text{nat}}}$.

1.3 Innate effectors

Apart from macrophages and dendritic cells, *Natural killers* (NK) are major effectors of the innate response. Their main immune functions are the induction of the infected cell cytolysis and IFNγ synthesis [6,15,22,23]. As the APC, they are recruited to the infection site by pro-inflammatory cytokines in synergy with IL12 [15,23,24]. Their proliferation and immune functions are activated by IFNγ and IL12, whereas IL10 inhibits the natural killer differentiation and their immune functions [22].

We represented the dynamics of activated NK (assuming that naive NK are not limiting) and included the regulations by the most influential cytokines. The recruitment of natural killers from the bloodstream (rate α_{NK}) requires pro-inflammatory cytokines and IL12 acting in synergy. Natural killers are then activated by IFNγ and IL12, whereas IL10 inhibits their activation. They are subject to natural death or/and migration (rate $\mu_{\text{NK}}^{\text{nat}}$).

Natural killers:

$$\begin{aligned}
\dot{\text{NK}} = &+ \alpha_{\text{NK}}\, \kappa^-(\text{IL10})\, \kappa^+(\text{IL12 Pi})\, \kappa^+(\text{IFN}\gamma + \text{IL12}) && \longleftarrow \text{recruitment \& activation} \\
&- \mu_{\text{NK}}^{\text{nat}}\, \text{NK} && \longleftarrow \text{decay}
\end{aligned} \tag{A5-5}$$

1.4 Adaptive effectors

Adaptive effectors consists of helper T cells, which orientate the adaptive response towards the cellular, humoral or regulatory response, as well as cytotoxic T cells. Only the broad lines of their dynamics are presented here (more details in [2, chap. 1]), based on the model proposed by Yates *et al.* for the regulation of helper T cell populations [25].

Helper T cells Naive helper T cells are activated by activated APC *via* a major histocompatibility complex (MHC). As PRRSv replication in APC down-regulates the expression of MHC [7–9], we distinguished the activation rates by T_m (rate $\alpha_E^{T_m}$) and T_i (rate $\alpha_E^{T_i}$). We assumed that the naive helper T cells are not limiting, so we did not represent their dynamics in the model.

Depending on the cytokine environment, activated helper T cells differentiate into three CD_4^+ T lymphocyte subtypes [6, 24–31]:

- *humoral effectors* (E_h), representing the type 2 helper T cells (T_{h2}), which is the default subtype, amplified by IL4 ;
- *cellular effectors* (E_c), representing type 1 helper T cells (T_{h1}), induced by IL12, amplified by IFNγ and inhibited by IL6;
- *regulatory effectors* (E_r), representing the regulatory T cells (T_{reg}), induced by TGFβ and inhibited by IL6.

In the model, IL6 is represented within the pro-inflammatory cytokine group. The proportion π_{E_k} of each effector E_k is hence defined as follows:

$$\begin{cases}
\pi_{E_h} = \dfrac{1 + \text{IL4} + \text{Pi}}{1 + \text{IL12} + \text{IFN}\gamma + \text{IL4} + \text{Pi} + \text{TGF}\beta} \\
\pi_{E_c} = \dfrac{\text{IL12} + \text{IFN}\gamma}{1 + \text{IL12} + \text{IFN}\gamma + \text{IL4} + \text{Pi} + \text{TGF}\beta} \\
\pi_{E_r} = \dfrac{\text{TGF}\beta}{1 + \text{IL12} + \text{IFN}\gamma + \text{IL4} + \text{Pi} + \text{TGF}\beta}
\end{cases}$$

Then activated helper T cells proliferate (rate p_E) and this proliferation is amplified by IL12 and inhibited by TGFβ [24]. Finally, these cells are subject to natural decay (rate μ_E^{nat}) amplified by TNFα, which induces their apoptosis [29–31].

Cellular effectors:

$$\begin{aligned}
\dot{E_c} = &+ (\alpha_E^{T_m}\, T_m + \alpha_E^{T_i}\, T_i)\, \frac{\mathrm{IL12} + \mathrm{IFN}\gamma}{1 + \mathrm{IL12} + \mathrm{IFN}\gamma + \mathrm{IL4} + \mathrm{Pi} + \mathrm{TGF}\beta} && \longleftarrow \text{activation \& differentiation} \\
&+ p_E\, E_c\, \kappa^-(\mathrm{TGF}\beta)\, [1 + \kappa^+(\mathrm{IL12})] && \longleftarrow \text{proliferation} \\
&- \mu_E^{\mathrm{nat}}\, E_c\, [1 + \kappa^+(\mathrm{TNF}\alpha)] && \longleftarrow \text{decay \& apoptosis}
\end{aligned} \tag{A5-6}$$

Humoral effectors:

$$\begin{aligned}
\dot{E_h} = &+ (\alpha_E^{T_m}\, T_m + \alpha_E^{T_i}\, T_i)\, \frac{1 + \mathrm{IL4} + \mathrm{Pi}}{1 + \mathrm{IL12} + \mathrm{IFN}\gamma + \mathrm{IL4} + \mathrm{Pi} + \mathrm{TGF}\beta} && \longleftarrow \text{activation \& differentiation} \\
&+ p_E\, E_h\, \kappa^-(\mathrm{TGF}\beta)\, [1 + \kappa^+(\mathrm{IL12})] && \longleftarrow \text{proliferation} \\
&- \mu_E^{\mathrm{nat}}\, E_h\, [1 + \kappa^+(\mathrm{TNF}\alpha)] && \longleftarrow \text{decay \& apoptosis}
\end{aligned} \tag{A5-7}$$

Regulatory effectors:

$$\begin{aligned}
\dot{E_r} = &+ (\alpha_E^{T_m}\, T_m + \alpha_E^{T_i}\, T_i)\, \frac{\mathrm{TGF}\beta}{1 + \mathrm{IL12} + \mathrm{IFN}\gamma + \mathrm{IL4} + \mathrm{Pi} + \mathrm{TGF}\beta} && \longleftarrow \text{activation \& differentiation} \\
&+ p_E\, E_r\, \kappa^-(\mathrm{TGF}\beta)\, [1 + \kappa^+(\mathrm{IL12})] && \longleftarrow \text{proliferation} \\
&- \mu_E^{\mathrm{nat}}\, E_r\, [1 + \kappa^+(\mathrm{TNF}\alpha)] && \longleftarrow \text{decay \& apoptosis}
\end{aligned} \tag{A5-8}$$

Cytotoxic T cells Also known as *Cytotoxic T Lymphocytes* (CTL), they are effectors of the adaptive response [3, 29–34]. They are responsible for the synthesis of antiviral cytokines IFNγ and TNFα and they induce the cytolysis of infected cells. We represented the dynamics of activated CTL, assuming that naive CTL are not limiting. We did not explicitly represent the licensing step, but the CTL activation (rate α_c) requires activated APC (mature T_m or infected T_i) and cellular effector (E_c). Just as for helper T cells, activation is followed by proliferation (rate p_E) and natural decay (rate μ_E^{nat}).

$$\begin{aligned}
\dot{\mathrm{CTL}} = &+ (\alpha_{\mathrm{CTL}}^{T_m}\, \frac{T_m}{1 + T_m} + \alpha_{\mathrm{CTL}}^{T_i}\, \frac{T_i}{1 + T_i})\, E_c && \longleftarrow \text{activation} \\
&+ p_E\, \mathrm{CTL}\, \kappa^-(\mathrm{TGF}\beta)\, [1 + \kappa^+(\mathrm{IL12})] && \longleftarrow \text{proliferation} \\
&- \mu_E^{\mathrm{nat}}\, \mathrm{CTL}\, [1 + \kappa^+(\mathrm{TNF}\alpha)] && \longleftarrow \text{decay \& apoptosis}
\end{aligned} \tag{A5-9}$$

1.5 Neutralising antibodies

Naive B cells are activated in the presence of the virus and helper T cells. They become *plasma cells*, which produce IgM, IgG, or IgA antibodies (or immunoglobulin Ig), depending on the helper T cell subtype [2,29–31]. Antibodies can either mark the pathogen to enhance its recognition by the immune system, or directly block its pathogenesis. Despite their high synthesis rate, antibodies with marking functions are inefficient for PRRSv infection resolution [35]. Therefore, we only considered *neutralising antibodies* in the model, which potentially regulate the infection [12,36,37]. As neutralising antibodies belong to the three isotypes, we did not differentiate the IgA-, IgG- and IgM-producing plasma cells.

Plasma Cells As for the adaptive effectors, the dynamics of plasma cells consists of three steps: activation (rate α_B^E), proliferation (rate p_B) and decay (rate μ_B^{nat}). The first step corresponds to the activation of naive B cells, which requires helper T cells ($E_\bullet$) and free viral particles (V) with a limiting effect of helper T cells for high viral concentrations. The proliferation is inhibited by TGFβ.

$$\begin{aligned}
\dot{B} = &+ \alpha_B^E\, \frac{V}{1 + V}\, (E_c + E_h + E_r) && \longleftarrow \text{activation} \\
&+ p_B\, B\, \kappa^-(\mathrm{TGF}\beta) && \longleftarrow \text{proliferation} \\
&- \mu_B^{\mathrm{nat}}\, B && \longleftarrow \text{decay}
\end{aligned} \tag{A5-10}$$

Neutralising antibodies They are synthesised by the plasma cells (synthesis rate ρ_{nAb}^B) and undergo natural decay (rate $\mu_{\mathrm{nAb}}^{\mathrm{nat}}$). The neutralisation rate of the virus (μ_V^{ad}) of the antibodies ($u_V^{\mathrm{nAb}}\, \mu_V^{\mathrm{ad}}$): parameter u_V^{nAb} accounts for stoichiometry (units antibody number required to neutralise a viral particle) and units (antibodies and virus concentrations are measured in different units).

$$
\begin{aligned}
\dot{\mathrm{nAb}} = &+ \rho^{B}_{\mathrm{nAb}}\, B && \longleftarrow \text{synthesis by plasma cells} \\
&- u^{\mathrm{nAb}}_{V}\, \mu^{\mathrm{ad}}_{V}\, \mathrm{nAb}\, V && \longleftarrow \text{neutralisation} \\
&- \mu^{\mathrm{nat}}_{\mathrm{nAb}}\, \mathrm{nAb} && \longleftarrow \text{decay}
\end{aligned} \qquad \text{(A5-11)}
$$

1.6 Cytokines

Cytokines are small proteins produced by activated immune cells that play a key role in cell-signalling. The major cytokine regulations and syntheses are represented in the model.

1.6.1 Cytokine regulations

Many processes driving the state variable (immune cells and cytokines) dynamics are regulated by a complex cytokine feedback system: activated immune cells synthesise cytokines, which up- and/or down-regulate the cell functions. Cytokines are recognised by specific receptors on the cell surface, inducing cascaded reactions within the cells. The higher the cytokine concentration, the stronger the effect. However, there is a limited number of receptors, so the effect saturates above a given cytokine concentration.

We formalised the cytokine up- (κ^+) and down-regulations (κ^-) based on a Michaelis–Menten function of the cytokine concentration (C_i) [38–40] as follows:

$$
\kappa^+(C_i) = \frac{v_m\, C_i}{k_m + C_i}\,, \qquad \kappa^-(C_i) = \frac{k_m}{k_m + C_i}\,,
$$

where v_m denotes the saturation factor and k_m the half saturation constant.

A cytokine can have three possible effects on a given basic rate (r):

- activation: $r\,\kappa^+(C_i)$, the basic rate increases with the cytokine concentration from 0 to $r\,v_m$;
- amplification: $r\,[1 + \kappa^+(C_i)]$, the basic rate increases from r to $r\,(1 + v_m)$;
- inhibition: $r\,\kappa^-(C_i)$, the basic rate decreases from r to zero.

Regulations often involve several cytokines (C_i and C_j) which can act

- either independently: $\kappa^{\pm}(C_i + C_j)$;
- or in synergy:
  - $\kappa^+(C_i\, C_j) = \frac{v_m\, C_i\, C_j}{k_m^2 + C_i\, C_j}$ for an activation or an amplification,
  - $\kappa^-(C_i\, C_j) = \frac{k_m^2}{k_m^2 + C_i\, C_j}$ for an inhibition.

As very few studies estimate the regulation parameters (k_m & v_m) in the literature [40], we used the same parameter values for all cytokine regulations.

1.6.2 Cytokine dynamics

Among the numerous cytokines involved in the immune response, we only considered the regulations by the eight most influential cytokines, including a functional group. They are listed below according to their function, some cytokines having dual functions:

- pro-inflammatory cytokines: Pi (grouping IL1β, IL6, IL8 and CCL2) and TNFα;
- antiviral cytokines: TNFα, IFNα (innate antiviral cytokines) and IFNγ (adaptive antiviral cytokine);
- immuno-modulatory cytokines: IL10 and TGFβ;
- immuno-regulatory cytokines: IFNγ, IL12, IL4, IL6 and TGFβ; where IFNγ and IL12 are pro-cellular cytokines, IL4 and IL6 are pro-humoral cytokines and TGFβ is a pro-regulatory cytokine.

The main cytokine regulations included in the model are summarised in Table A5-2; cells involved in their synthesis are summarised in Table A5-3.

We assumed that cytokines are efficient enough to neglect their consumption when they interact with a cell. So the cytokine dynamics results from their synthesis by immune cells (rates ρ^y_x, where $_x$ denotes the cytokine and ^{y} the synthesising cell) and their natural decay (rate μ_C).

Pro-inflammatory cytokines They amplify the recruitment of innate immune cells (APC and natural killers) [13–15,34]. Moreover, IL6 blocks the differentiation towards the cellular and regulatory adaptive effectors [29–31].

Table A5-2. Cytokine regulations. Main positive (+) and negative (–) regulations included in the model for various immune mechanisms.

	Cytokines							
	Pi*	TNFα	IFNα	IFNγ	IL12	IL4	IL10	TGFβ
Innate response								
APC recruitment	+				+			
NK recruitment & activation	+			+	+		–	
APC apoptosis		+						
Phagocytosis		+	+	+			–	–
APC permissiveness		–	–	–			+	–
Viral replication		–	–	–				
Adaptive response								
Humoral response	+			–	–	+	+	–
Cellular response	–			+	+	–	–	–
Regulatory response	–			–	–	–	±	+
Cytokine syntheses								
Pro-inflammatory IL1β							–	
IL6, IL8	+	+						
Innate antiviral TNFα							–	
Adaptive antiviral IFNγ		+	+	+	+		–	–
Immuno-regulatory IL12							–	
Immuno-modulatory IL10							+	±
Specific references	[13–15, 34, 41]	[3, 13, 16, 32, 41–45]		[3, 32–34]	[46]	[29–31]	[7, 8, 14, 41, 47–49]	[50]
Global references				[6, 20, 24, 26–31, 51, 52]				

* Pro-inflammatory cytokine group Pi include: IL1β, IL6, IL8, CCL2

Table A5-3. Cytokine syntheses. Production of cytokines included (✓) in the model by innate or adaptive immune cells.

	Cytokines								
	Pi*	TNFα	IFNα	IFNγ	IL12	IL4	IL10	TGFβ	References
Innate cells									
Activated APC	✓	✓	✓		✓		✓		[15, 53]†
Activated natural killers				✓					[15, 23]†
Adaptive cells									
Humoral effectors						✓	✓		†
Cellular effectors				✓					†
Regulatory effectors							✓	✓	†
Cytotoxic T Lymphocytes		✓		✓					†

* Pro-inflammatory cytokine group Pi include: IL1β, IL6, IL8, CCL2
† Global references: [6, 20, 24, 26–28, 30, 54, 55]

They are synthesised by activated APC (T_m and T_i). The synthesis of IL1β is inhibited by IL10 [52], whereas the synthesis of IL6 and IL8 is co-activated by IL1β and TNFα [14,15,34].

$$\begin{aligned} \dot{\text{Pi}} = &+ \rho_{\text{Pi}}^{T}\, \kappa^{-}(\text{IL10})\, \kappa^{+}(\text{Pi TNF}\alpha)\, (T_m + T_i) &\longleftarrow \text{synthesis} \\ &- \mu_c^{\text{nat}}\, \text{Pi} &\longleftarrow \text{decay} \end{aligned} \tag{A5-12}$$

Antiviral cytokines They promote the phagocytosis and reduce the infection by inhibiting the permissiveness of macrophages and conventional dendritic cells and the viral replication. The synthesis of these cytokines differ.

TNFα is synthesised by activated APC (T_m and T_i) and cytotoxic T cells (CTL), these syntheses being inhibited by IL10 [49].

$$\begin{aligned} \dot{\text{TNF}\alpha} = &+ \left(\rho_{\text{TNF}\alpha}^{T}\, (T_m + T_i) + \rho_{\text{TNF}\alpha}^{\text{CTL}}\, \text{CTL}\right)\, \kappa^{-}(\text{IL10}) &\longleftarrow \text{synthesis} \\ &- \mu_c^{\text{nat}}\, \text{TNF}\alpha &\longleftarrow \text{decay} \end{aligned} \tag{A5-13}$$

TNFα also induces the apoptosis of APC and T cells.

IFNα is synthesised by infected cells (T_i) and mature plasmacytoid dendritic cells (in T_m).

$$\begin{aligned} \dot{\text{IFN}\alpha} = &+ \rho_{\text{IFN}\alpha}^{T}\, (T_m + T_i) &\longleftarrow \text{synthesis} \\ &- \mu_c^{\text{nat}}\, \text{IFN}\alpha &\longleftarrow \text{decay} \end{aligned} \tag{A5-14}$$

IFNγ is synthesised by activated conventional dendritic cells (in T_m, T_i), natural killers (NK), type 1 helper T cells (E_c) and cytotoxic T cells (CTL). These syntheses are auto-amplified and inhibited by TGFβ and IL10.

$$\begin{aligned} \dot{\text{IFN}\gamma} = &+ \left(\rho_{\text{IFN}\gamma}^{T}\, (T_m + T_i) + \rho_{\text{IFN}\gamma}^{\text{NK}}\, \text{NK} + \rho_{\text{IFN}\gamma}^{E_c,\text{CTL}}\, (E_c + \text{CTL})\right)\, \kappa^{-}(\text{TGF}\beta + \text{IL10})\, [1 + \kappa^{+}(\text{IFN}\gamma)] &\longleftarrow \text{synthesis} \\ &- \mu_c^{\text{nat}}\, \text{IFN}\gamma &\longleftarrow \text{decay} \end{aligned} \tag{A5-15}$$

IFNγ is also an immuno-regulatory cytokine and it orientates the adaptive response towards the cellular response [3,29–34].

Immuno-modulatory cytokines Their functions are globally the oppposit of the antiviral cytokine functions and they inhibit numerous immune mechanisms.

IL10 inhibits the natural killer activation and the phagocytosis, amplifies the target cell permissiveness and inhibits the synthesis of numerous cytokines [49].

It is synthesised by activated target cells and regulatory T cells (E_r), both syntheses being amplified by TGFβ. It is also synthesised by humoral effectors (E_h), this synthesis being auto-amplified and inhibited by TGFβ.

$$\begin{aligned} \dot{\text{IL10}} = &+ \left(\rho_{\text{IL10}}^{T}\, (T_m + T_i) + \rho_{\text{IL10}}^{E_r}\, E_r\right)\, [1 + \kappa^{+}(\text{TGF}\beta)] \\ &+ \rho_{\text{IL10}}^{E_h}\, E_h\, \kappa^{-}(\text{TGF}\beta)\, [1 + \kappa^{+}(\text{IL10})] &\longleftarrow \text{synthesis} \\ &- \mu_c^{\text{nat}}\, \text{IL10} &\longleftarrow \text{decay} \end{aligned} \tag{A5-16}$$

TGFβ inhibits the phagocytosis, the target cell permissiveness, the lymphocyte proliferation, as well as the synthesis of IFNγ and IL10 by humoral effectors. It also induces the differentiation of helper T cells towards regulatory effectors.

TGFβ is synthesised by activated conventional dendritic cells (in T_m, T_i) and regulatory effectors (E_r).

$$\begin{aligned} \dot{\text{TGF}\beta} = &+ \rho_{\text{TGF}\beta}^{T}\, (T_m + T_i) + \rho_{\text{TGF}\beta}^{E_r}\, E_r &\longleftarrow \text{synthesis} \\ &- \mu_c^{\text{nat}}\, \text{TGF}\beta &\longleftarrow \text{decay} \end{aligned} \tag{A5-17}$$

Immuno-regulatory cytokines Immuno-regulatory cytokines consist of IFNγ (see antiviral), IL12, IL4, IL6 (see pro-inflammatory) and TGFβ (see immuno-modulatory). They exhibit various functions [24,26–31], in particular the regulation of the adaptive immune response. They also regulate the recruitment of macrophages and natural killers, the phagocytosis and infection, as well as cytokine syntheses. They are produced by cells of the innate and adaptive response.

IL12 co-amplifies the recruitment of innate cells, activates the natural killers, induces the differentiation of helper T cells towards cellular effectors and amplifies the proliferation of T cells.

It is synthesised by activated macrophages and conventional dendritic cells (in T_m, T_i), as well as cytotoxic T cells (CTL). IL10 inhibits the synthesis by macrophages, IFNγ amplifies the synthesis by dendritic cells.

$$\begin{aligned} \dot{\text{IL12}} = &+ \rho_{\text{IL12}}^{T} \, (T_m + T_i) \, \kappa^{-}(\text{IL10}) \, [1 + \kappa^{+}(\text{IFN}\gamma)] + \rho_{\text{IL12}}^{\text{CTL}} \, \text{CTL} && \longleftarrow \textsf{synthesis} \\ &- \mu_c^{\text{nat}} \, \text{IL12} && \longleftarrow \textsf{decay} \end{aligned} \tag{A5-18}$$

IL4 amplifies the differentiation of helper T cells towards humoral effectors (E_h), which synthesise this cytokine.

$$\begin{aligned} \dot{\text{IL4}} = &+ \rho_{\text{IL4}}^{E_h} \, E_h && \longleftarrow \textsf{synthesis} \\ &- \mu_c^{\text{nat}} \, \text{IL4} && \longleftarrow \textsf{decay} \end{aligned} \tag{A5-19}$$

Table A5-4. Model parameters.

PARAM.	DESCRIPTION	VALUE(S)	UNIT	REF.
Target cell – PRRSv interactions				
η_T	phagocytosis by T_n and T_m	$[10^{-13} - 10^{-8}]$	$[V]^{-1}$ day^{-1}	[2]
γ_T	activation loss of T_m	0.1	day^{-1}	[40, 56]
β_T	infection of T_n and T_m	$[10^{-13} - 10^{-8}]$	$[V]^{-1}$ day^{-1}	[2]
e_T	excretion by T_i	$[10^{5} - 10^{8}]$	[V] $[Ce]^{-1}$ day^{-1}	[56]
Recruitment, activation and proliferation rates				
R_T	recruitment of T_n on the infection place	$5\,10^3$	[Ce] day^{-1}	[39, 57, 58]
α_{NK}	activation of NK	$5\,10^3$	[Ce] day^{-1}	[39]
$\alpha_E^{T_m}$	activation of helper T cells by T_m	$[0.01 - 10]$	day^{-1}	[40, 59]
$\alpha_E^{T_i}$	activation of helper T cells by T_i	$\alpha_E^{T_m} \times 10^{-2}$	day^{-1}	[2]
$\alpha_{\mathrm{CTL}}^{T_m}$	activation of cytotoxic T cells by T_m	$\alpha_E^{T_m} \times 10$	$[Ce]^{-1}$ day^{-1}	[2]
$\alpha_{\mathrm{CTL}}^{T_i}$	activation of cytotoxic T cells by T_i	$\alpha_E^{T_m} \times 0.1$	$[Ce]^{-1}$ day^{-1}	[2]
α_B^{E}	activation of plasma cells by helper T cells	$[10^{-3} - 10]$	$[V]^{-1}$ day^{-1}	[2]
p_E	proliferation of T cells	$\mu_E^{\mathrm{nat}} \times 0.1$	day^{-1}	[58, 59]
p_B	proliferation of plasma cells	$\mu_B^{\mathrm{nat}} \times 0.1$	day^{-1}	[60]
Antibody and cytokine synthesis rates				
ρ_{nAb}^{B}	neutralising antibody synthesis by plasma cells	$[10^{-3} - 10]$	[Ig] $[Ce]^{-1}$ day^{-1}	[56, 60]
ρ_{Pi}^{T}	Pi synthesis by T_m and T_i	$[0 - 10^{4}]$	[Cy] $[Ce]^{-1}$ day^{-1}	[2]
$\rho_{\mathrm{TNF}\alpha}^{T}$	TNFα synthesis by T_m and T_i	$[0 - 10^{4}]$	[Cy] $[Ce]^{-1}$ day^{-1}	[39, 59]
$\rho_{\mathrm{TNF}\alpha}^{\mathrm{CTL}}$	TNFα synthesis by CTL	1	[Cy] $[Ce]^{-1}$ day^{-1}	[59]
$\rho_{\mathrm{IFN}\alpha}^{T}$	IFNα synthesis by T_m and T_i	$[0 - 10^{4}]$	[Cy] $[Ce]^{-1}$ day^{-1}	[2]
$\rho_{\mathrm{IFN}\gamma}^{T}$	IFNγ synthesis by T_m and T_i	$[0 - 10^{4}]$	[Cy] $[Ce]^{-1}$ day^{-1}	[2]
$\rho_{\mathrm{IFN}\gamma}^{\mathrm{NK}}$	IFNγ synthesis by NK	1	[Cy] $[Ce]^{-1}$ day^{-1}	[2]
$\rho_{\mathrm{IFN}\gamma}^{E_c,\mathrm{CTL}}$	IFNγ synthesis by E_c and CTL	1	[Cy] $[Ce]^{-1}$ day^{-1}	[40, 58, 59]
ρ_{IL12}^{T}	IL12 synthesis by T_m and T_i	$[0 - 10^{4}]$	[Cy] $[Ce]^{-1}$ day^{-1}	[39, 40, 58, 59]
$\rho_{\mathrm{IL12}}^{\mathrm{CTL}}$	IL12 synthesis by CTL	1	[Cy] $[Ce]^{-1}$ day^{-1}	[2]
$\rho_{\mathrm{IL4}}^{E_h}$	IL4 synthesis by E_h	$[0 - 10^{4}]$	[Cy] $[Ce]^{-1}$ day^{-1}	[40, 59]
ρ_{IL10}^{T}	IL10 synthesis by T_m and T_i	$[0 - 10^{4}]$	[Cy] $[Ce]^{-1}$ day^{-1}	[39, 40]
$\rho_{\mathrm{IL10}}^{E_r}$	IL10 synthesis by E_r	1	[Cy] $[Ce]^{-1}$ day^{-1}	[40, 58, 59]
$\rho_{\mathrm{IL10}}^{E_h}$	IL10 synthesis by E_h	1	[Cy] $[Ce]^{-1}$ day^{-1}	[2]
$\rho_{\mathrm{TGF}\beta}^{T}$	TGFβ synthesis by T_m and T_i	$[0 - 10^{4}]$	[Cy] $[Ce]^{-1}$ day^{-1}	[2]
$\rho_{\mathrm{TGF}\beta}^{E_r}$	TGFβ synthesis by E_r	1	[Cy] $[Ce]^{-1}$ day^{-1}	[2]
Mortality/migration rates				
μ_T^{nat}	natural decay of target cells	0.2	day^{-1}	[39, 40, 58, 60]
μ_T^{ap}	apoptosis of target cells by TNFα	5	day^{-1}	[58, 59]
μ_T^{inn}	cytolysis of T_i by NK	0.1	$[Ce]^{-1}$ day^{-1}	[58]
μ_T^{ad}	cytolysis of T_i by CTL	10	$[Ce]^{-1}$ day^{-1}	[40, 56]
δ_μ	increased mortality of T_i	1.1	∅	[2]
μ_V^{nat}	natural decay of viral particles	0.6	day^{-1}	[56]
μ_V^{ad}	neutralisation of viral particles by antibodies	10^{-3}	day^{-1}	[2]
$\mu_{\mathrm{NK}}^{\mathrm{nat}}$	natural decay of NK	0.06	day^{-1}	[2]
μ_E^{nat}	natural decay of T cells	0.08	day^{-1}	[39, 40, 58, 59]
μ_B^{nat}	natural decay of plasma cells	0.04	day^{-1}	[60]
$\mu_{\mathrm{nAb}}^{\mathrm{nat}}$	natural decay of antibodies	0.03	day^{-1}	[56, 60]
μ_c^{nat}	natural decay of cytokines	20	day^{-1}	[40, 59]
Cytokine regulation functions κ				
v_m	saturation factor (cytokine regulations)	1.5	∅	[2]
k_m	half saturation constant (cytokine regulations)	30	[Cy]	[2]
Misc				
u_T^{V}	target cell – virus unit conversion	1	[V] $[Ce]^{-1}$	[2]
u_V^{nAb}	virus – antibody unit conversion	10	[Ig] $[V]^{-1}$	[56]
Viral exposure (bell-shaped curve)				
$\mathcal{N}_E$	total exposure dose	10^{5}	[V]	[61]
D_E	exposure duration	5	days	[2]
a_E	shape parameter	3	∅	[2]
b_E	shape parameter	3	∅	[2]

VALUE(S): either fixed or estimated from a given [range]

UNIT: cells [Ce] = cells/ml, virus [V] = $TCID_{50}$/ml, cytokines [Cy] = pg/ml, antibodies [Ig] = pg/ml, none ∅

REF.: bibliographic references from experimental studies and modelling approaches

2 Sensitivity analyses

Our aim was to identify and estimate parameters or mechanisms that characterise the uniphasic and biphasic viremia profiles. Global Sensitivity Analyses (GSA) were hence performed to identify the most influential parameters on the viral dynamics.

We used a method adapted to multivariate outputs, such as the viral dynamics, based on factorial design, principal component analysis (PCA) and analysis of variance (ANOVA). This method is implemented in the `R` package `multisensi`[1]. Each analysis consists of four steps.

1. Definition of the parameter space: setting the parameters that vary and their ranges, based on the literature knowledge and previous work [1, 62].
2. Definition of the fractional factorial design of numerical experiments: selecting the combinations of parameter (= factor) values (= levels) to be tested. We used `R` package `planor`[2] to generate the design. `planor` optimizes the number of input combinations with regards to an ANOVA model, minimizing the computation effort and is well suited to compute the sensitivity indices [63]. Compared to random sampling based methods, `planor` is consequently more suitable to high dimensional problems characterized by evenly influential parameters with strong interactions (such as ours, see results below).
3. Exploration of the parameter space: simulating the model for the parameter sets defined in the previous step, to obtain a set of outputs (the viral dynamics here).
4. Computation of the sensitivity indices (GSI): proportion of multivariate output variance explained by each parameter or interaction between parameters. First, a Principal Component Analysis (PCA) on the model multivariate output is performed, followed by an ANOVA-based sensitivity analyses on each of the first principal components of the PCA. Indeed, one assumes that an ANOVA "metamodel" (possibly with interactions) links the inputs to the output score (projection) on each principal component. Finally, the Generalized Sensitivity Indices (GSI) are computed, corresponding to the weighted means of the sensitivity indices over the first principal components.

2.1 First analysis: influence of all model parameters on the viral dynamics

Parameter space We first investigated the influence of 46 out of the 49 model parameters on the viral dynamics. The unit conversion parameters (u_T^V, u_{nAb}^V) were fixed and the total exposure dose ($\mathcal{N}_E$) was set to the inoculation dose. Parameter ranges were defined based on our previous work [1] and additional literature (*cf.* Table A5-4), especially for the adaptive immune mechanisms added to our previous model. Rough explorations of the parameter space were performed, varying the parameters one at a time, to ensure that all mechanisms had a variable and realistic influence on the model outputs (visual inspection of the viral and immune dynamics).

Design We chose three levels for each of the 46 parameter (Figure A5-1**A.**). A full factorial design would result in far too many simulations ($3^{46} \simeq 10^{22}$) and a design built to estimate all main factorial effects and two-factor interactions (*i.e.* resolution V design) would still be too big for a preliminary analysis (*ca.* 10^4 simulations). So we built a fractional factorial design to estimate the main effect of each parameter unconfounded by two-factor interactions (*i.e.* resolution IV design), which resulted in 729 parameter sets.

Results

- The resulting viral dynamics (Figure A5-1**B.**) exhibited a wide variability, including realistic and unrealistic behaviours (very fast resolution or persistence), showing that the parameter space covers more than the observed variability.
- 93% of the variance of the resulting viral dynamics was explained by the statistical model linking the viral dynamics to the parameters. Almost all parameters had comparable contributions to this variance, ranging between less than 4% of this variance (Figure A5-1**C.**). So we could not identify a "reasonable" subset of parameters with a marked influence of the viral dynamics (*i.e.* consisting of less than 20 parameters).

[1]Bidot C., Lamboni M., Monod H. (2017). multisensi: Multivariate Sensitivity Analysis. R package version 2.1, `https://CRAN.R-project.org/package=multisensi`

[2]Monod H., Bouvier A., Kobilinsky A. (2017). planor: Generation of Regular Factorial Designs. R package version 1.3-7, `https://CRAN.R-project.org/package=planor`

Figure A5-1. Global sensitivity analysis to assess the influence of model parameters on the viral dynamics. A. Parameter levels for the 46 model parameters that varied. **B.** Viremia for the 729 parameter sets obtained from a resolution IV fractional factorial design, built with the parameter levels given in **A.** using R package `planor`[2]. **C.** Generalised sensitivity indices (GSI), representing the contribution of each parameter to the viral dynamics variance, computed using R package `multisensi`[1]. 93% of the variance (R^2) was explained by the statistical model linking the viral dynamics to the parameters.

A. parameter levels

parameter	levels
η_T	$[10^{-13}\,;\,10^{-10.5}\,;\,10^{-8}]$
γ_T	$[0.05\,;\,0.1\,;\,0.15]$
β_T	$[10^{-13}\,;\,10^{-10.5}\,;\,10^{-8}]$
e_T	$[10^{5}\,;\,10^{7}\,;\,10^{8}]$
R_T	$[10^{4}\,;\,10^{5}\,;\,10^{6}]$
α_{NK}	$[10^{3}\,;\,5\,10^{3}\,;\,10^{4}]$
$\alpha_E^{T_m}$	$[0.01\,;\,10^{-0.5}\,;\,10]$
$\alpha_E^{T_i}$	$\alpha_E^{T_m} \times [10^{-3}\,;\,10^{-2}\,;\,10^{-1}]$
$\alpha_{\text{CTL}}^{T_m}$	$\alpha_E^{T_m} \times [5\,;\,10\,;\,15]$
$\alpha_{\text{CTL}}^{T_i}$	$\alpha_{\text{CTL}}^{T_m} \times [10^{-3}\,;\,10^{-2}\,;\,10^{-1}]$
α_B^E	$[10^{-3}\,;\,0.1\,;\,10]$
p_E	$[0.005\,;\,0.008\,;\,0.011]$
p_B	$[0.001\,;\,0.004\,;\,0.007]$
ρ_{nAb}^B	$[10^{-3}\,;\,0.1\,;\,10]$
ρ_{Pi}^T	$[0\,;\,10\,;\,10^{4}]$
$\rho_{\text{TNF}\alpha}^T$	$[0\,;\,10\,;\,10^{4}]$
$\rho_{\text{TNF}\alpha}^{\text{CTL}}$	$[0.1\,;\,1\,;\,10]$
$\rho_{\text{IFN}\alpha}^T$	$[0\,;\,10\,;\,10^{4}]$
$\rho_{\text{IFN}\gamma}^T$	$[0\,;\,10\,;\,10^{4}]$
$\rho_{\text{IFN}\gamma}^{\text{NK}}$	$0.1\,;\,1\,;\,10]$
$\rho_{\text{IFN}\gamma}^{E_c,\text{CTL}}$	$[0.1\,;\,1\,;\,10]$
ρ_{IL12}^T	$[0\,;\,10\,;\,10^{4}]$
$\rho_{\text{IL12}}^{\text{CTL}}$	$[0.1\,;\,1\,;\,10]$
$\rho_{\text{IL4}}^{E_h}$	$[0\,;\,10\,;\,10^{4}]$
ρ_{IL10}^T	$[0\,;\,10\,;\,10^{4}]$
$\rho_{\text{IL10}}^{E_r}$	$[0.1\,;\,1\,;\,10]$
$\rho_{\text{IL10}}^{E_h}$	$[0.1\,;\,1\,;\,10]$
$\rho_{\text{TGF}\beta}^T$	$[0\,;\,10\,;\,10^{4}]$
$\rho_{\text{TGF}\beta}^{E_r}$	$[0.1\,;\,1\,;\,10]$
μ_T^{nat}	$[0.1\,;\,0.2\,;\,0.3]$
μ_T^{ap}	$[1\,;\,5\,;\,10]$
μ_T^{inn}	$[0.01\,;\,0.1\,;\,11]$
μ_T^{ad}	$[5\,;\,10\,;\,15]$
δ_μ	$[1\,;\,1.1\,;\,1.2]$
μ_V^{nat}	$[0.5\,;\,0.6\,;\,0.7]$
μ_V^{ad}	$[10^{-4}\,;\,10^{-3}\,;\,10^{-2}]$
$\mu_{\text{NK}}^{\text{nat}}$	$[0.03\,;\,0.06\,;\,0.09]$
μ_E^{nat}	$[0.05\,;\,0.08\,;\,0.11]$
μ_B^{nat}	$[0.001\,;\,0.04\,;\,0.07]$
$\mu_{\text{nAb}}^{\text{nat}}$	$[0.01\,;\,0.03\,;\,0.05]$
μ_c^{nat}	$[10\,;\,20\,;\,30]$
v_m	$[1\,;\,1.5\,;\,2]$
k_m	$[20\,;\,30\,;\,40]$
D_E	$[3\,;\,5\,;\,7]$
a_E	$[2.5\,;\,3\,;\,3.5]$
b_E	$[2.5\,;\,3\,;\,3.5]$

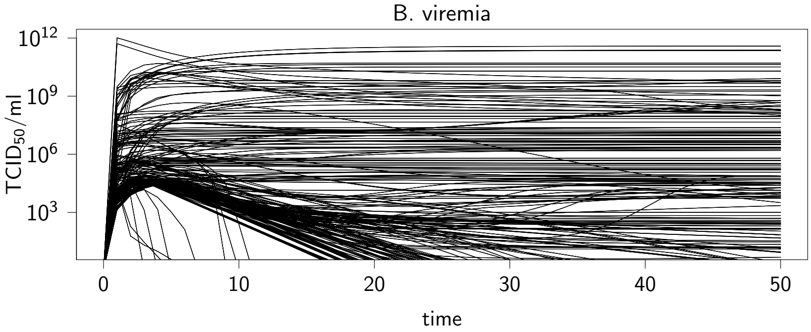

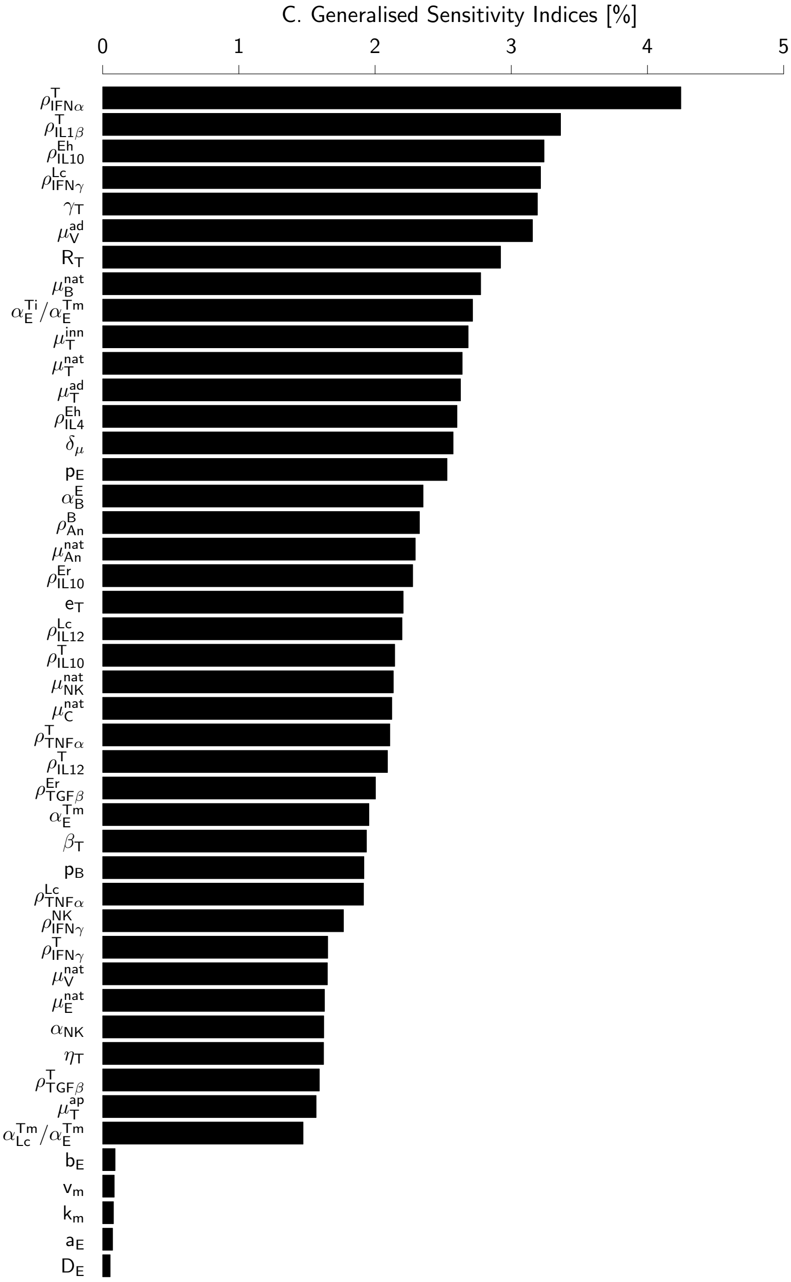

2.2 Second analysis: influence of the estimated parameters on the viral dynamics

Parameter selection As the first analysis could not identify a parameter subset with a marked influence on the viral dynamics, we chose the parameters to be estimated for each viremia profile based on biological knowledge. We selected parameters linked to between-host susceptibility: (i) the host–virus interactions (β_T, η_T) and the virus capacity to replicate (e_T), (ii) the activation $(\alpha_E^{T_m}, \alpha_B^E, \rho_{\mathrm{nAb}}^B)$ and orientation (linked to cytokine synthesis rates) of the adaptive response, (iii) the host capacity to synthesise antiviral $(\rho_{\mathrm{TNF}\alpha}^T, \rho_{\mathrm{IFN}\alpha}^T, \rho_{\mathrm{IFN}\gamma}^T)$ *vs* immuno-modulatory cytokines $(\rho_{\mathrm{IL}10}^T, \rho_{\mathrm{TGF}\beta}^T)$ [12, 21, 64–67].

We only varied the cytokine synthesis rates by activated target cells ($\rho_\bullet^T$, but not by other effectors (except for IL4 for which no information was found), as studies hypothesise that between-host variability of the immune response is mostly due to cascaded reactions initiated by the virus–target cell interactions [12, 45, 67, 68]. The relative activation rates of the adaptive response depending on $\alpha_E^{T_m}$ were fixed, so the actual rates varied with $\alpha_E^{T_m}$ (*e.g.* $\alpha_E^{T_i} = 10^{-2}\alpha_E^{T_m}$). Mortality rates were fixed, assuming that apoptosis, cytolysis and virus neutralisation varied according to the concentrations of TNFα, natural killers plus cytotoxic cells and neutralising antibodies, respectively.

We hence obtained 14 parameters to be estimated and we set the remaining parameters to their intermediate value.

Design For each of the 14 parameters, we used the same three levels as defined in the first analysis (Figure A5-2**A.**). We built a design to estimate all main factorial effects and two-factor interactions unconfounded by two-factor interactions (*i.e.* resolution V design), which resulted in 2187 parameter sets.

Results

- As in the first analysis, the resulting viral dynamics (Figure A5-2**B.**) exhibited a wide variability.
- 64% of the variance of the resulting viral dynamics was explained by the statistical model linking the viral dynamics to the parameters. Each parameter main effect contributed to less than 2% of this variance, but interaction contributions were high (Figure A5-2**C**). The rates of TGFβ, IFNγ and IL12 had a fairly negligible impact; all other parameters contributed to ±8% of the viral dynamics variance.

Conclusion From these analyses, we could not single out parameters or mechanisms with a major impact on the viral dynamics. However, we made sure that the parameter ranges chosen here and used for the parameter estimation could generate very diverse viral dynamics, realistic and unrealistic (very fast resolution or persistence), showing that the parameter space covers more than the observed variability.

Figure A5-2. Global sensitivity analysis to assess the influence of estimated parameters on the viral dynamics. A. Parameter levels for the 14 model parameters that varied. **B.** Viral dynamics for the 2187 parameter sets obtained from a resolution V fractional factorial design, built with the parameter levels given in **A** using `R` package `planor`[2]. **C.** Generalised sensitivity indices (GSI), decomposed into main effect (black bar) and interaction (grey bar), representing the contribution of each parameter to the viral dynamics variance, computed using `R` package `multisensi`[1]. 64% of the variance (R^2) was explained by the statistical model linking the viral dynamics to the parameters.

A. parameter levels

parameter	levels
η_{T}	$[10^{-13}; 10^{-10.5}; 10^{-8}]$
β_{T}	$[10^{-13}; 10^{-10.5}; 10^{-8}]$
$\mathsf{e_T}$	$[10^{5}; 10^{7}; 10^{8}]$
$\alpha_{\mathsf{E}}^{\mathsf{Tm}}$	$[10^{-2}; 10^{-0.5}; 10]$
$\alpha_{\mathsf{B}}^{\mathsf{E}}$	$[10^{-3}; 10^{-1}; 10]$
$\rho_{\mathsf{An}}^{\mathsf{B}}$	$[10^{-3}; 10^{-1}; 10]$
$\rho_{\mathsf{Pi}}^{\mathsf{T}}$	$[0; 10; 10^{4}]$
$\rho_{\mathsf{TNF}\alpha}^{\mathsf{T}}$	$[0; 10; 10^{4}]$
$\rho_{\mathsf{IFN}\alpha}^{\mathsf{T}}$	$[0; 10; 10^{4}]$
$\rho_{\mathsf{IFN}\gamma}^{\mathsf{T}}$	$[0; 10; 10^{4}]$
$\rho_{\mathsf{IL12}}^{\mathsf{T}}$	$[0; 10; 10^{4}]$
$\rho_{\mathsf{IL4}}^{\mathsf{Eh}}$	$[0; 10; 10^{4}]$
$\rho_{\mathsf{IL10}}^{\mathsf{T}}$	$[0; 10; 10^{4}]$
$\rho_{\mathsf{TGF}\beta}^{\mathsf{T}}$	$[0; 10; 10^{4}]$

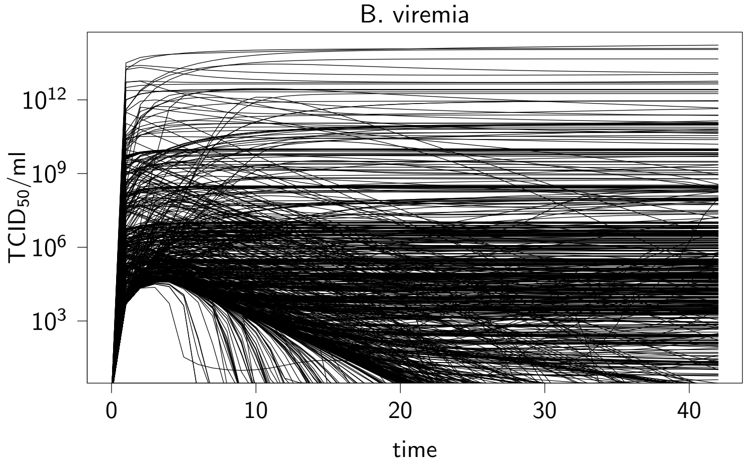

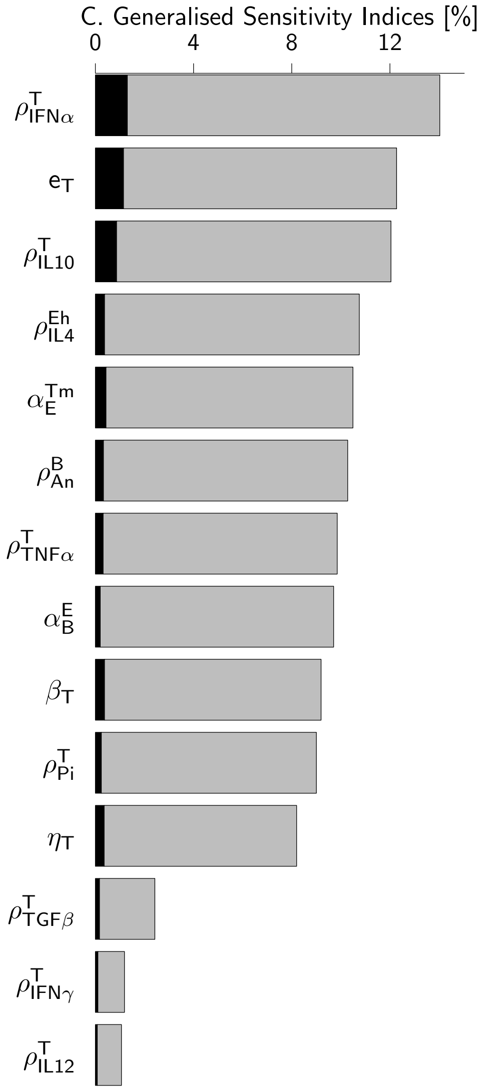

References

1. Go N, Bidot C, Belloc C, Touzeau S. Integrative model of the immune response to a pulmonary macrophage infection: what determines the infection duration? PLoS ONE. 2014;9(9):e107818. doi:10.1371/journal.pone.0107818.

2. Go N. Modelling the immune response to the Porcine Reproductive and Respiratory Syndrome virus [PhD Thesis]. AgroParisTech (ED ABIES). Paris, France; 2014. Available from: `https://hal.inria.fr/tel-01100983/`.

3. Zimmerman J, Benfield DA, Murtaugh MP, Osorio F, Stevenson GW, Torremorell M. Porcine reproductive and respiratory syndrome virus (porcine arterivirus). In: Straw BE, Zimmerman JJ, D'Allaire S, Taylor DL, editors. Diseases of swine. ninth ed. Oxford, UK: Blackwell; 2006. p. 387–418.

4. Baumann A, Mateu E, Murtaugh MP, Summerfield A. Impact of genotype 1 and 2 of porcine reproductive and respiratory syndrome viruses on interferon-α responses by plasmacytoid dendritic cells. Vet Res. 2013;44(1):33. doi:10.1186/1297-9716-44-33.

5. Silva-Campa E, Cordoba L, Fraile L, Flores-Mendoza L, Montoya M, Hernández J. European genotype of porcine reproductive and respiratory syndrome (PRRSV) infects monocyte-derived dendritic cells but does not induce Treg cells. Virology. 2010;396(2):264–271. doi:10.1016/j.virol.2009.10.024.

6. Rouse BT, Sehrawat S. Immunity and immunopathology to viruses: what decides the outcome? Nat Rev Immunol. 2010;10(7):514–526. doi:10.1038/nri2802.

7. Chang HC, Peng YT, Chang HL, Chaung HC, Chung WB. Phenotypic and functional modulation of bone marrow-derived dendritic cells by porcine reproductive and respiratory syndrome virus. Vet Microbiol. 2008;129(3–4):281–293. doi:10.1016/j.vetmic.2007.12.002.

8. Flores-Mendoza L, Silva-Campa E, Reséndiz M, Osorio FA, Hernández J. Porcine reproductive and respiratory syndrome virus infects mature porcine dendritic cells and up-regulates interleukin-10 production. Clin Vaccine Immunol. 2008;15:720–725. doi:10.1128/CVI.00224-07.

9. Wang X, Eaton M, Mayer M, Li H, He D, Nelson E, et al. Porcine reproductive and respiratory syndrome virus productively infects monocyte-derived dendritic cells and compromises their antigen-presenting ability. Arch Virol. 2007;152(2):289–303. doi:10.1007/s00705-006-0857-1.

10. Park JY, Kim HS, Seo SH. Characterization of interaction between porcine reproductive and respiratory syndrome virus and porcine dendritic cells. J Microbiol Biotechn. 2008;18(10):1709–1716.

11. Silva-Campa E, Flores-Mendoza L, Reséndiz M, Pinelli-Saavedra A, Mata-Haro V, Mwangi W, et al. Induction of T helper 3 regulatory cells by dendritic cells infected with porcine reproductive and respiratory syndrome virus. Virology. 2009;387(2):373–379. doi:10.1016/j.virol.2009.02.033.

12. Kimman TG, Cornelissen LA, Moormann RJ, Rebel JMJ, Stockhofe-Zurwieden N. Challenges for porcine reproductive and respiratory syndrome virus (PRRSV) vaccinology. Vaccine. 2009;27(28):3704–3718. doi:10.1016/j.vaccine.2009.04.022.

13. Van Reeth K, Van Gucht S, Pensaert M. In vivo studies on cytokine involvement during acute viral respiratory disease of swine: troublesome but rewarding. Vet Immunol Immunop. 2002;87(3-4):161–168. doi:10.1016/S0165-2427(02)00047-8.

14. Roth AJ, Thacker EL. Immune system. In: Straw BE, Zimmerman JJ, D'Allaire S, Taylor DL, editors. Diseases of swine. ninth ed. Oxford, UK: Blackwell; 2006. p. 15–36.

15. Tosi MF. Innate immune responses to infection. J Allergy Clin Immun. 2005;116(2):241–249. doi:10.1016/j.jaci.2005.05.036.

16. Choi C, Chae C. Expression of tumour necrosis factor alpha is associated with apoptosis in lungs of pigs experimentally infected with porcine reproductive and respiratory syndrome virus. Res Vet Sci. 2002;72(1):45–49. doi:10.1053/rvsc.2001.0519.

17. Thanawongnuwech R. Pulmonary intravascular macrophages (PIMs): What do we know about their role in PRRSV infection in pigs. Wetchasan Sattawaphaet (The Thai journal of veterinary medicine). 1999;29.

18. Takeda K, Kaisho T, Akira S. Toll-like receptors. Annu Rev Immunol. 2003;21(1):335–376. doi:10.1146/annurev.immunol.21.120601.141126.

19. Cafruny WA, Duman RG, Wong GH, Said S, Ward-Demo P, Rowland RR, et al. Porcine reproductive and respiratory syndrome virus (PRRSV) infection spreads by cell-to-cell transfer in cultured MARC-145 cells, is dependent on an intact cytoskeleton, and is suppressed by drug-targeting of cell permissiveness to virus infection. Virol J. 2006;3:90. doi:10.1186/1743-422X-3-90.

20. Darwich L, Díaz I, Mateu E. Certainties, doubts and hypotheses in porcine reproductive and respiratory syndrome virus immunobiology. Virus Res. 2010;154(1-2):123–132. doi:10.1016/j.virusres.2010.07.017.

21. Thanawongnuwech R, Suradhat S. Taming PRRSV: Revisiting the control strategies and vaccine design. Virus Res. 2010;154(1-2):133–140. doi:10.1016/j.virusres.2010.09.003.

22. DeFranco AL, Locksley RM, Robertson M, Cunin R. Immunité : la réponse immunitaire dans les maladies infectieuses et inflammatoires. 2nd ed. Bruxelles, Belgium: DeBoeck; 2009.

23. Vidal SM, Khakoo SI, Biron CA. Natural killer cell responses during viral infections: flexibility and conditioning of innate immunity by experience. Curr Opin Virol. 2011;1(6):497–512. doi:10.1016/j.coviro.2011.10.017.

24. Borghetti P. Cell-mediated immunity and viral infection in pig. In: PRRS fatti vs speculazioni. Parma, Italy: Università degli Studi di Parma, Dipartimento di Salute Animale; 2005. p. 27–46. Available from: `http://medvet.unipr.it/cgi-bin/campusnet/documenti.pl/Show?_id=7d0d`.

25. Yates A, Bergmann C, Van Hemmen JL, Stark J, Callard R. Cytokine-modulated regulation of helper T cell populations. J Theor Biol. 2000;206(4):539–560. doi:10.1006/jtbi.2000.2147.

26. Bosch AATM, Biesbroek G, Trzcinski K, Sanders EAM, Bogaert D. Viral and bacterial interactions in the upper respiratory tract. PLoS Pathog. 2013;9(1):e1003057. doi:10.1371/journal.ppat.1003057.

27. Braciale TJ, Sun J, Kim TS. Regulating the adaptive immune response to respiratory virus infection. Nat Rev Immunol. 2012;12(4):295–305. doi:10.1038/nri3166.

28. Coquerelle C, Moser M. DC subsets in positive and negative regulation of immunity. Immunol Rev. 2010;234(1):317–334. doi:10.1111/j.0105-2896.2009.00887.x.

29. Kidd P. Th1/Th2 balance: the hypothesis, its limitations, and implications for health and disease. Altern Med Rev. 2003;8(3):223–246.

30. LeRoith T, Ahmed SA. Regulatory T cells and viral disease. In: Khatami M, editor. Inflammation, Chronic Diseases and Cancer: Cell and Molecular Biology, Immunology and Clinical Bases. Rijeka, Croatia: InTech; 2012. p. 121–144.

31. Knosp CA, Johnston JA. Regulation of CD4+ T-cell polarization by suppressor of cytokine signalling proteins. Immunology. 2012;135(2):101–111. doi:10.1111/j.1365-2567.2011.03520.x.

32. Lopez-Fuertes L, Campos E, Domenech N, Ezquerra A, Castro JM, Dominguez J, et al. Porcine reproductive and respiratory syndrome (PRRS) virus down-modulates TNF-α production in infected macrophages. Virus Res. 1999;69(269):41–46. doi:10.1016/S0168-1702(00)00172-6.

33. Molina RM, Cha SH, Chittick W, Lawson S, Murtaugh MP, Nelson EA, et al. Immune response against porcine reproductive and respiratory syndrome virus during acute and chronic infection. Vet Immunol Immunop. 2008;126:283–292. doi:10.1016/j.vetimm.2008.08.002.

34. Lunney JK, Fritz ER, Reecy JM, andr Elizabeth Prucnal DK, Molina R, Christopher-Hennings J, et al. Interleukin-8, interleukin-1β, and interferon-γ levels are linked to PRRS virus clearance. Viral Immunol. 2010;23(2):127–134. doi:10.1089/vim.2009.0087.

35. Yoon KJ, Wu LL, Zimmerman JJ, Platt KB. Field isolates of porcine reproductive and respiratory syndrome virus (PRRSV) vary in their susceptibility to antibody dependent enhancement (ADE) of infection. Vet Microbiol. 1997;55(1):277–287. doi:10.1016/S0378-1135(96)01338-7.

36. Delputte PL, Meerts P, Costers S, Nauwynck HJ. Effect of virus-specific antibodies on attachment, internalization and infection of porcine reproductive and respiratory syndrome virus in primary macrophages. Vet Immunol Immunop. 2004;102(3):179–188. doi:10.1016/j.vetimm.2004.09.007.

37. Ostrowski M, Galeota JA, Jar AM, Platt KB, Osorio FA, Lopez OJ. Identification of neutralizing and nonneutralizing epitopes in the porcine reproductive and respiratory syndrome virus GP5 ectodomain. J Virol. 2002;76(9):4241–4250. doi:10.1128/JVI.76.9.4241-4250.2002.

38. Gammack D, Ganguli S, Marino S, Segovia-Juarez J, Kirschner D. Understanding the immune response in tuberculosis using different mathematical models and biological scales. Multiscale Model Sim. 2005;3(2):312–345. doi:10.1137/040603127.

39. Marino S, Myers A, Flynn JL, Kirschner DE. TNF and IL-10 are major factors in modulation of the phagocytic cell environment in lung and lymph node in tuberculosis: A next-generation two-compartmental model. J Theor Biol. 2010;265(4):586–598. doi:10.1016/j.jtbi.2010.05.012.

40. Wigginton JE, Kirschner D. A model to predict cell-mediated immune regulatory mechanisms during human infection with Mycobacterium tuberculosis. J Immunol. 2001;166:1951–1967. doi:10.4049/jimmunol.166.3.1951.

41. Peng YT, Chaung HC, Chang HL, Chang HC, Chung WB. Modulations of phenotype and cytokine expression of porcine bone marrow-derived dendritic cells by porcine reproductive and respiratory syndrome virus. Vet Microbiol. 2009;136(3-4):359–365. doi:10.1016/j.vetmic.2008.11.013.

42. Albina E. Le point sur le dernier-né des arterivirus : le virus du syndrome dysgénésique et respiratoire porcin (SDRP). Virologie. 2000;4(2):113–121.

43. Murtaugh MP. PRRSV/host interaction. In: PRRS fatti vs speculazioni. Parma, Italy: Università degli Studi di Parma, Dipartimento di Salute Animale; 2005. p. 73–80. Available from: `http://medvet.unipr.it/cgi-bin/campusnet/documenti.pl/Show?_id=7d0d`.

44. Miller LC, Laegreid WW, Bono JL, Chitko-McKown CG, Fox JM. Interferon type I response in porcine reproductive and respiratory syndrome virus-infected MARC-145 cells. Arch Virol. 2004;149:2453–2463. doi:10.1007/s00705-004-0377-9.

45. Gimeno M, Darwich L, Díaz I, de la Torre E, Pujols J, Martin M, et al. Cytokine profiles and phenotype regulation of antigen presenting cells by genotype-I porcine reproductive and respiratory syndrome virus isolates. Vet Res. 2011;42(9). doi:10.1186/1297-9716-42-9.

46. Thanawongnuwech R, Rungsipipat A, Disatian S, Saiyasombat R, Napakanaporn S, Halbur PG. Immunohistochemical staining of IFN_γ positive cells in porcine reproductive and respiratory syndrome virus-infected lungs. Vet Immunol Immunop. 2003;91(1):73–77. doi:10.1016/S0165-2427(02)00268-4.

47. Yoo D, Song C, Sun Y, Du Y, Kim O, Liu HC. Modulation of host cell responses and evasion strategies for porcine reproductive and respiratory syndrome virus. Virus Res. 2010;154(1–2):48–60. doi:10.1016/j.virusres.2010.07.019.

48. Suradhat S, Thanawongnuwech R, Poovorawan Y. Upregulation of IL-10 gene expression in porcine peripheral blood mononuclear cells by porcine reproductive and respiratory syndrome. J Gen Virol. 2003;84:453–459. doi:10.1099/vir.0.18698-0.

49. Moore KW, de Waal Malefyt R, Coffman RL, O'Garra A. Interleukin-10 and the interleukin-10 receptor. Annu Rev Immunol. 2001;19(1):683–765. doi:10.1146/annurev.immunol.19.1.683.

50. Gómez-Laguna J, Salguero FJ, Pallarés FJ, Carrasco L. Immunopathogenesis of porcine reproductive and respiratory syndrome in the respiratory tract of pigs. Vet J. 2013;195(2):148–155. doi:10.1016/j.tvjl.2012.11.012.

51. Murtaugh MP, Xiao Z, Zuckermann F. Immunological responses of swine to porcine reproductive and respiratory syndrome virus infection. Viral Immunol. 2002;15(4):533–547. doi:10.1089/088282402320914485.

52. Gómez-Laguna J, Salguero FJ, Barranco I, Pallares FJ, Rodriguez-Gomez IM, Bernabe A, et al. Cytokine expression by macrophages in the lung of pigs infected with the porcine reproductive and respiratory syndrome virus. J Comp Pathol. 2009;142(1):51–60. doi:10.1016/j.jcpa.2009.07.004.

53. Underhill DM, Goodridge HS. Information processing during phagocytosis. Nat Rev Immunol. 2012;12:492–502. doi:10.1038/nri3244.

54. Akira S, Takeda K, Kaisho T. Toll-like receptors: critical proteins linking innate and acquired immunity. Nat Immunol. 2001;2(8):675–680. doi:10.1038/90609.

55. Sen GC. Viruses and interferons. Annu Rev Microbiol. 2001;55:255–281. doi:10.1146/annurev.micro.55.1.255.

56. Hancioglu B, Swigon D, Clermont G. An ensemble model of the human immune response to influenza A virus infection and its application to the evaluation of treatment strategies; 2014.

57. Labarque GG, Nauwynck HJ, Van Reeth K, Pensaert MB. Effect of cellular changes and onset of humoral immunity on the replication of porcine reproductive and respiratory syndrome virus in the lungs of pigs. J Gen Virol. 2000;81(5):1327–1334. doi:10.1099/0022-1317-81-5-1327.

58. Marino S, Kirschner DE. The human immune response to Mycobacterium tuberculosis in lung and lymphe node. J Theor Biol. 2004;227:463–486. doi:10.1016/j.jtbi.2003.11.023.

59. Sud D, Bigbee C, Flynn JL, Kirschner DE. Contribution of CD8+ T cells to control of Mycobacterium tuberculosis infection. J Immunol. 2006;176(7):4296–4314. doi:10.4049/jimmunol.176.7.4296.

60. Lee HY, Topham DJ, Park SY, Hollenbaugh J, Treanor J, Mosmann TR, et al. Simulation and prediction of the adaptive immune response to influenza A virus infection. J Virol. 2009;83(14):7151–7165. doi:10.1128/JVI.00098-09.

61. Rowland RRR, Lunney J, Dekkers J. Control of porcine reproductive and respiratory syndrome (PRRS) through genetic improvements in disease resistance and tolerance. Front Genet. 2012;3:260. doi:10.3389/fgene.2012.00260.

62. Go N, Belloc C, Bidot C, Touzeau S. Why, when and how should exposure be considered at the within-host scale? A modelling contribution to PRRSv infection. Mathematical Medicine and Biology: a journal of the IMA. 2018;doi:10.1093/imammb/dqy005.

63. Monod H, Bouvier A. Construction and randomization of regular factorial designs with the R package planor; 2016.

64. Nauwynck HJ, Van Gorp H, Vanhee M, Karniychuk U, Geldhof M, Cao A, et al. Micro-dissecting the pathogenesis and immune response of PRRSV infection paves the way for more efficient PRRSV vaccines. Transbound Emerg Dis. 2012;59:50–54. doi:10.1111/j.1865-1682.2011.01292.x.

65. Murtaugh MP, Genzow M. Immunological solutions for treatment and prevention of porcine reproductive and respiratory syndrome (PRRS). Vaccine. 2011;29(46):8192–8204. doi:10.1016/j.vaccine.2011.09.013.

66. Darwich L, Gimeno M, Sibila M, Díaz I, de la Torre E, Dotti S, et al. Genetic and immunobiological diversities of porcine reproductive and respiratory syndrome genotype I strains. Vet Microbiol. 2011;150(1–2):49–62. doi:10.1016/j.vetmic.2011.01.008.

67. Lunney JK, Chen H. Genetic control of host resistance to porcine reproductive and respiratory syndrome virus (PRRSV) infection. Virus Res. 2010;154(1–2):161–169. doi:10.1016/j.virusres.2010.08.004.

68. Ait-Ali T, Wilson AD, Carré W, Westcott DG, Frossard JP, Mellencamp MA, et al. Host inhibits replication of European porcine reproductive and respiratory syndrome virus in macrophages by altering differential regulation of type-I interferon transcriptional responses. Immunogenetics. 2011;63(7):437–448. doi:10.1007/s00251-011-0518-8.
